# Achromatic metasurfaces by dispersion customization for ultra-broadband acoustic beam engineering

**DOI:** 10.1093/nsr/nwac030

**Published:** 2022-02-24

**Authors:** Hao-Wen Dong, Chen Shen, Sheng-Dong Zhao, Weibao Qiu, Hairong Zheng, Chuanzeng Zhang, Steven A Cummer, Yue-Sheng Wang, Daining Fang, Li Cheng

**Affiliations:** Institute of Advanced Structure Technology, Beijing Institute of Technology, Beijing 100081, China; Department of Mechanical Engineering, The Hong Kong Polytechnic University, Hong Kong, China; Department of Electrical and Computer Engineering, Duke University, Durham, NC 27708, USA; Department of Mechanical Engineering, Rowan University, Glassboro, NJ 08028, USA; School of Mathematics and Statistics, Qingdao University, Qingdao 266071, China; Paul C. Lauterbur Research Center for Biomedical Imaging, Institute of Biomedical and Health Engineering, Shenzhen Institutes of Advanced Technology, Chinese Academy of Sciences, Shenzhen 518055, China; Paul C. Lauterbur Research Center for Biomedical Imaging, Institute of Biomedical and Health Engineering, Shenzhen Institutes of Advanced Technology, Chinese Academy of Sciences, Shenzhen 518055, China; Department of Civil Engineering, University of Siegen, D-57068 Siegen, Germany; Department of Electrical and Computer Engineering, Duke University, Durham, NC 27708, USA; Deparment of Mechanics, School of Mechanical Engineering, Tianjin University, Tianjin 300350, China; Institute of Advanced Structure Technology, Beijing Institute of Technology, Beijing 100081, China; Department of Mechanical Engineering, The Hong Kong Polytechnic University, Hong Kong, China

**Keywords:** ultra-broadband metasurfaces, achromatic, inverse design, customized dispersion, multiple scattering

## Abstract

Metasurfaces, the ultra-thin media with extraordinary wavefront modulation ability, have shown great promise for many potential applications. However, most of the existing metasurfaces are limited by narrow-band and strong dispersive modulation, which complicates their real-world applications and, therefore require strict customized dispersion. To address this issue, we report a general methodology for generating ultra-broadband achromatic metasurfaces with prescribed ultra-broadband achromatic properties in a bottom-up inverse-design paradigm. We demonstrate three ultra-broadband functionalities, including acoustic beam deflection, focusing and levitation, with relative bandwidths of 93.3%, 120% and 118.9%, respectively. In addition, we reveal a relationship between broadband achromatic functionality and element dispersion. All metasurface elements have anisotropic and asymmetric geometries with multiple scatterers and local cavities that synthetically support internal resonances, bi-anisotropy and multiple scattering for ultra-broadband customized dispersion. Our study opens new horizons for ultra-broadband highly efficient achromatic functional devices, with promising extension to optical and elastic metamaterials.

## Teaser

This paper reports inversely designed metasurfaces with customized dispersion through bottom-up topology optimization to realize frequency-independent and ultra-broadband acoustic functionalities including directional energy transmission and convergence and ultrasound particle levitation.

## INTRODUCTION

Metasurfaces have offered tremendous possibility in the fields of optical devices [1–9], carpet cloaking [9], medical ultrasound [10,11], architectural acoustics [12] and non-destructive testing [13]. Acoustic metasurfaces (AMs) [10–12,14–24], as an important category of metamaterials, have revolutionized the way they control the absorption, reflection and transmission of acoustic waves due to their extraordinary wavefront-shaping ability. Examples include a near-perfect sound absorber [15], noise control [14,22], Schroeder diffuser [12], cloaking [16], beam deflection [10,11,20,23], focusing [18,23,24], asymmetric transmission [19], vortex beam [17] and acoustic levitation [11]. Regardless of the specific functionality, the core of the metasurface design is to create elements that have a unique refractive index and impedance to control the amplitude and phase shift of transmitted or reflected waves. The existing metasurfaces of airborne sound are mostly based on Helmholtz-resonator [12,16–20,23], space-coiling [10,11,14,24] or membrane-type [15] elements. However, the vast majority of AMs suffer from narrow bandwidth and dispersive functionality, leaving little to no use for real-world applications. Although coding [18] or tunable [24] approaches can, in principle, increase bandwidth by adjusting the element configuration, they are limited by the apparent dispersion in functionality and the increased complexity and cost of the system, which make the devices impractical. Moreover, the tunable metasurface strategy is highly frequency-dependent. While functionality can be guaranteed at discrete frequencies, it is unlikely to accommodate broadband incident wave packets containing multiple frequencies simultaneously.

To achieve an arbitrarily defined broadband functionality, each metasurface element must provide the required local amplitude and/or phase modulation, which is controlled over the entire operating bandwidth. This modulation depends on the specific functionality and leads to the customization of the dispersion of each element. Recent studies have shown that broadband [4–7] optical multi-wavelength achromatic [25] devices are achievable using sophisticated artificial structures [4–7,25]. However, their broadband performance still relies on the achromatic phase modulation of elements [4–7,25] rather than on completely arbitrary dispersion, taking into account the constituent elements and whole metasurface of the macrostructure. In this case, the terminology of the so-called broadband is different from what is here stated as achromatic. Consequently, in the areas of both optical metasurfaces and AMs, most of the existing studies have focused on various wave functionalities and phenomena with the inevitable strong dispersions. From an application perspective, an ultra-broadband frequency-independent feature with relative bandwidths of >100% is still missing, especially for metasurfaces. Using its unique ability to simultaneously process the geometric and physical properties of a structure in a large search space, the inverse-design methodology based on topology optimization offers an ideal tool for creating new metasurface features with distinct characteristics. To achieve this, the most important task is to identify the relationship between broadband achromatic functionality and the dispersion of elements, and simultaneously construct the desired microscopic and macroscopic dispersions.

To address the aforementioned challenges, we propose a systematic bottom-up inverse-design methodology for implementing ultra-broadband and customized metasurfaces, and explore the physics behind different functionalities. Our study demonstrates the feasibility of developing a systematic inverse-design model for constructing ultra-broadband achromatic metasurfaces, the dispersions of which can be tactically tailored. Optimized elements can be either non-dispersive or dispersive through customization, exhibiting many asymmetric scatterers and cavities within the elements. By combining these elements into specific patterns, we consistently achieve a class of metasurfaces that entail ultra-broadband, achromatic beam deflection, focusing and levitation, beyond the relative bandwidth of existing achromatic metasurfaces. Finally, we also reveal the ultra-broadband mechanism that results from a combination of integrated internal resonances, bi-anisotropy and multiple-scattering effects. We design three types of achromatic metasurfaces with customized microscopic and macroscopic dispersions, which entail ultra-broadband frequency-independent acoustic beam functionalities that have not been achieved before. The discovered asymmetric and bi-anisotropic microstructural topological characteristic and synthetical ultra-broadband achromatic mechanism offer a new basic platform for designing broadband frequency-independent metasurfaces. Meanwhile, we demonstrate a metasurface that supports ultra-broadband, stable and single-sided ultrasound levitation that can hardly be achieved with traditional ultrasound levitation technologies.

The presented metasurfaces can simultaneously provide the customized specific dispersion of elements and non-dispersive nature of functionality within the ultra-broadband range, which leads to ultra-broadband achromatic metasurfaces, which, as expected, will allow them to be used in noise-control systems, medical ultrasonics and contactless particle control assembly. Meanwhile, the proposed ultra-broadband achromatic strategy can be extended to optical and elastic metamaterials for highly efficient frequency-independent functional devices.

## RESULTS AND DISCUSSION

**Figure 1.**
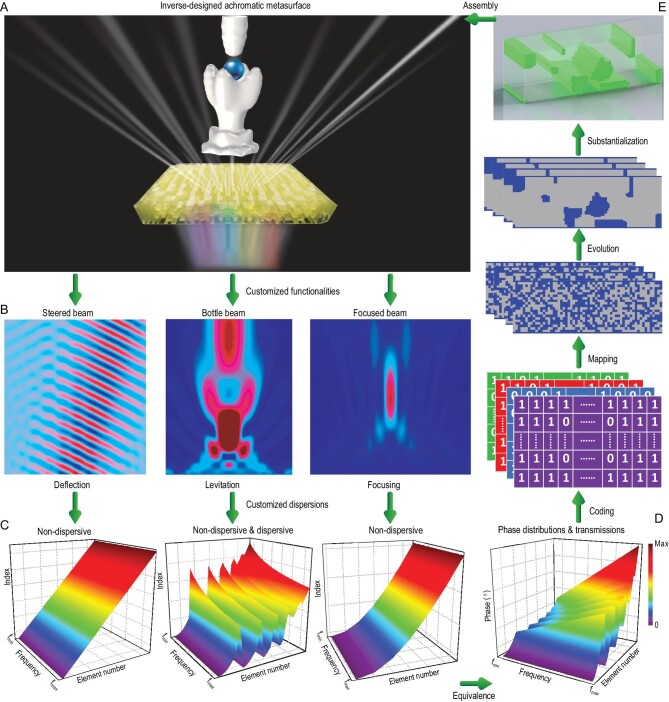
Scheme of ultra-broadband achromatic metasurfaces designed by bottom-up topology optimization. (A) Ultra-broadband achromatic metasurface (middle yellow block) that can transform incident acoustic waves of multi-wavelength into an exotic bottle beam with almost the same acoustic amplitude isosurface (two white stereoscopic objects) at different frequencies within [*f*_min_ and *f*_max_], making a particle suspended in the same location in the air. The different colors below the metasurface represent different operating frequencies/wavelengths. (B) Anomalous beam deflection, focusing and levitation by the metasurface. (C) The required effective indices of all metasurface elements for beam deflection (left), focusing (right) and levitation (middle) at (B). (D) Target phase distributions of elements for custom functionality. (E) Sketch of a heuristic bottom-up strategy. Gray and blue pixels represent air and solid, respectively.

Figure 1 shows a diagram of ultra-broadband metasurfaces consisting of inverse-designed elements. When waves strike the metasurface, achromatic functionality will be realized over a wide frequency range if all output wavefronts are identical at different frequencies (Fig. 1A). For example, this typical achromatic feature will generate a bottle beam with nearly the same focusing location at each operating frequency. Its particular acoustic amplitude isosurface can additionally provide push and pull forces of acoustic radiation to levitate a particle in the air in the ultra-broadband range. This ultra-broadband levitation phenomenon can find promising scientific and industrial applications in the fields of the control and assembly of non-contact particles/droplets/biological cells [26], even in 3D imaging techniques [26]. Inverse-designed ultra-broadband achromatic metasurfaces can also result in ultra-broadband customized achromatic functionalities such as anomalous beam deflection [10], focusing [10,11] and even levitation [11] (Fig. 1B) with beam characteristics on demand. In principle, broadband wavefront manipulation is associated with the specific dispersion of each element and the careful combination of all elements (Fig. 1C)—that is, the microscopic and macroscopic dispersions of metasurfaces. To steer the beam (Supplementary Text S1), every element must maintain a constant effective index over the frequency range. Since the required phase shift between adjacent elements increases linearly, the index for all elements also increases linearly at the same frequency. Likewise, for focusing (Supplementary Text S2), every element must maintain a constant index. On the other hand, the differences between the indices of adjacent elements vary depending on the position of the element, so that the same focus point can be achieved. However, for levitation (Supplementary Text S3), the elements must maintain a certain dispersion (in this case called a customized dispersion)—that is, the indices remain constant for some elements of the metasurface, and for others they decrease nonlinearly with increasing frequency. Variation of indices between different elements is different from the other two functionalities. Obviously, these complex and stringent dispersions, along with the complex phase shift of the elements (Fig. 1D), make the broadband design extremely challenging. To implement achromatic design, all elements at different locations on the metasurface must simultaneously satisfy specific effective refractive indices, relative group delays and relative group delay dispersions (Supplementary Text S4). Under the influence of the objective function and constraints for the bottom-up topology optimization [27–29] (Supplementary Text S5), the binary algorithm starts from a random initial population and gradually generates an optimized element by customization through a series of genetic operations and structural entropy filters. All optimized elements are assembled into a metasurface for a customized functionality. Note that the proposed optimization problem for ultra-broadband metasurfaces with customized dispersion is a new inverse-design problem in the wave field, which can be solved with other evolutionary algorithms, gradient algorithms and even machine learning.

Unlike traditional top-down topology-optimization approaches, the proposed bottom-up inverse-design paradigm is based on achromatic dispersion tailoring and offers the following salient advantages: (i) it is based on the responses of metasurface elements rather than the entire physical field, thus simultaneously reducing the search space, optimization complexity and computational cost; (ii) it avoids the blindness of the empirical design; (iii) its element-based design philosophy embraces and combines multiple physical mechanisms, thus maximizing the ultimate performance such as broadband, high efficiency and achromatism, etc.; and (iv) it reveals novel topological configurations of the elements with broadband features.

### Engineered metasurface for ultra-broadband achromatic beam deflection

**Figure 2.**
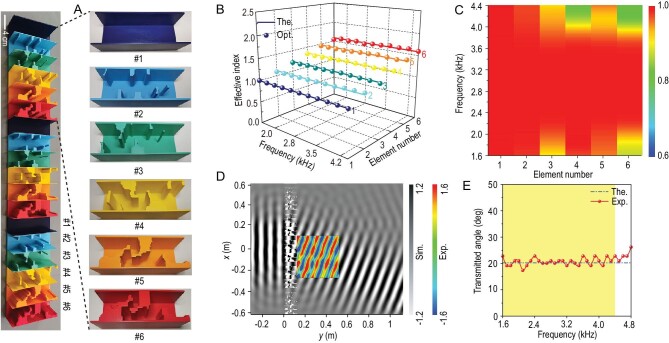
Ultra-broadband achromatic beam deflection using an inverse-designed metasurface. (A) Manufactured gradient metasurface with three periods, each containing six optimized elements. (B) and (C) Theoretical (The.), optimized (Opt.) effective indices (B) and transmission coefficients (C) of all optimized elements within [1600 Hz and 4400 Hz]. (D) Simulated acoustic pressure field at 4000 Hz. The inset shows the measured acoustic field, which is in good agreement with the calculated results. (E) Measured angle of transmission through the metasurface. The dash-and-dot line indicates the theoretical desired angle of refraction of 20.2° which is arbitrarily prescribed. The yellow shadow area represents the bandwidth of the metasurface within [1600 Hz and 4400 Hz]. The assumption of a hard-wall boundary is made due to negligible interaction between air and solid parts.

We start with anomalous refraction to validate the proposed inverse-design strategy. To achieve broadband negative refraction with a fixed angle of 20.2°, we perform topology optimization of 2D metasurface elements (Supplementary Text S9 and Supplementary Movies S6–S8) based on the generalized Snell's law [1]. As shown in Fig. 2A, with the exception of Element #1, all optimized elements have common topological features: (i) highly asymmetric geometry; (ii) complex curved air channels with non-uniform widths; and (iii) distributed cavities located in several local domains. In particular, Elements #5 and #6 have similar shapes, while Elements #2, #3 and #4 are different. This indicates that broadband non-dispersive properties can be achieved through an optimal combination of topologies rather than a single configuration. Previous design strategies using only a Helmholtz resonator or a spatial spiral topology [10–12,14,16–20,23,24] are unlikely to provide such broadband functionality. Topologically optimized structures have ultra-broadband constant effective indices (Fig. 2B). Note that the final operating range becomes [1600 Hz and 4400 Hz], although the target frequency range is [2000 Hz and 4000 Hz] when optimized. This distinct broadening effect implies relatively stable performance. Limiting the transmission also ensures that all elements provide a gain of a transmission coefficient in excess of 80% over almost the entire frequency band (Fig. 2C). As a result, the assembled metasurface clearly induces ultra-broadband anomalous refraction with required constant angle (Figs 2D and E), even when considering thermo-viscous loss [14,30] (Supplementary Text S10). Since the supercell (i.e. Elements #1–#6) cannot ensure full 2π phase coverage at all frequencies, the metasurface inevitably causes phase jump at the interface of two periods, causing some near-field scattering (Supplementary Text S10). In any case, the optimized asymmetric element provides a new platform for ultra-broadband beam deflection. For larger deflective angles, even close to 90°, the inverse-design model, through enlarging the design domain, can still lead to achromatic, high-efficiency and wide-angle deflection. Meanwhile, viscous-thermal effects can also be apprehended in the model to further improve the design accuracy.

### Engineered metasurfaces for ultra-broadband achromatic focusing

**Figure 3.**
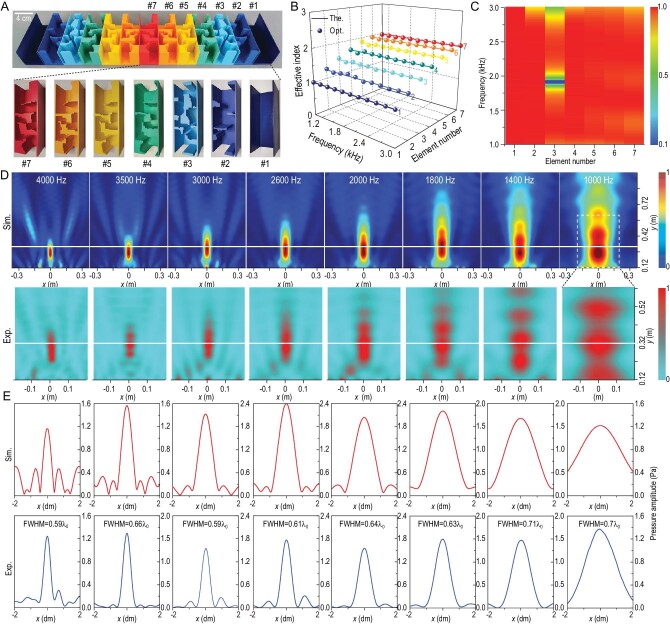
Ultra-broadband achromatic focusing using an inverse-designed metasurface. (A) Manufactured converging metasurface based on 13 optimized elements. (B) and (C) Theoretical (The.), optimized (Opt.) effective indices (B) and transmission coefficients (C) of all optimized elements within [1000 Hz and 3000 Hz]. (D) Simulated (Sim.) and measured (Exp.) acoustic amplitude fields at eight representative frequencies within [1000 Hz and 4000 Hz]. The white solid line indicates the desired focal depth (*F*_0_ = 0.2 m). The dashed line indicates the measured domain of 0.4 × 0.5 m. For the same frequency, the simulated and measured fields use different normalization scales, which represent the maximal pressure amplitudes. Different normalization scales are used for results at different frequencies. (E) Profiles of simulated (Sim.) and measured focusing fields (Exp.) in focal planes. The full widths at half maximum of the measured focus are also shown, where *λ*_0_ denotes the wavelength in air.

Here we use the aforementioned inverse-design strategy to systematically construct a metasurface for ultra-broadband focusing at a constant depth of focus (Fig. 3A). The optimized elements have the same topological characteristics as the elements for ultra-broadband beam deflection (Supplementary Text S11). Interestingly, Elements #4–#7 have similar overall topology—that is, four solid blocks asymmetrically distributed across the domain. Element #3 is different from the other six elements. But Elements #4–#7 have a very similar topology. This demonstrates that the configurations of Elements #4–#7 have reached the limit, i.e. one configuration cannot implement all the required indices. All optimized elements, except Element #1, are a combination of a helical structure and cavities. As shown in Fig. 3B, the optimized elements can indeed display constant indices over the entire frequency range, showing clear ultra-broadband non-dispersive properties while maintaining relatively high transmission (Fig. 3C). We notice a low transmission region for Element #3 around 2.0 kHz, which is likely caused by resonance associated with the relatively thin channels in the structure. However, this low transmission only occurs in a very narrow frequency range [1870 Hz and 1940 Hz] and the average transmission of Element #3 remains high. Simulated and measured acoustic fields in Fig. 3D clearly demonstrate the ultra-broadband achromatic performance in terms of focusing energy at the same focal depth. Moreover, the target range [1000 Hz and 3000 Hz] used in optimization is also extended to [1000 Hz and 4000 Hz], showing stable ultra-broadband properties. The observed focusing feature can also be maintained when the thermo-viscous losses are taken into account. In other words, thermal-viscous losses have a limited impact (Supplementary Text S12), although the focusing efficiency inevitably becomes somewhat lower. Consequently, an optimized asymmetrical topology can serve as an ideal candidate for ultra-broadband non-dispersive metasurface engineering.

### Engineered metasurface for ultra-broadband achromatic levitation

**Figure 4.**
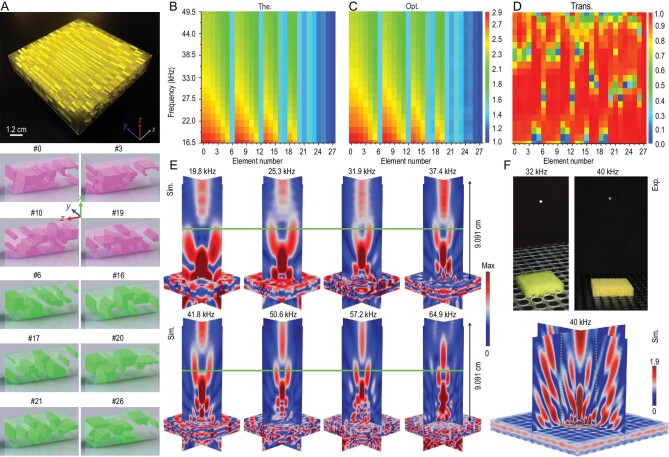
Ultra-broadband achromatic ultrasound levitation using an inverse-designed metasurface. (A) Manufactured micron-scale 3D metasurface (5.46 × 5.46 × 1.2 cm) with 13 × 13 optimized elements (4.2 mm × 4.2 mm × 1.2 cm). The metasurface has a square symmetry. The geometries of typical optimized elements with new topologies are presented below the metasurface. Pink and green graphs denote the representative non-linear dispersive and non-dispersive elements, respectively. (B)–(D) Theoretical (The.) (B) optimized (Opt.) (C) effective indices and transmission coefficients (Trans.) (D) of all optimized elements within [16.5 kHz and 49.5 kHz]. (E) Simulated (Sim.) bottle beam fields at eight representative frequencies within [16.5 kHz and 66 kHz]. The green solid line indicates a predetermined levitation location (*F*_0_ = 4.545 cm). (F) Measured (Exp.) stable single-sided levitation in air at two representative frequencies (32 and 40 kHz) of a red polystyrene bead with a diameter of 3.4 mm. In the simulation (Sim.) mimicking the experiment, a metasurface is directly placed on an array of ultrasonic sources, causing the desired bottle beam. The area within the green dashed lines represents the area covered by the metasurface.

As shown in Fig. 4, we apply our inverse-design strategy to implement ultra-broadband stable single-sided acoustic levitation by generating bottle beams [31,32]. A square-symmetrical metasurface is built to create the bottle beam (Fig. 4A and Supplementary Text S13). The optimized asymmetrical levitation elements are also more complex structurally than the topologies shown in Figs 2A and 3A. The calculated required effective indices of the elements show that some elements are non-dispersive, while others are highly dispersive (Fig. 4B). Figure 4C illustrates that the optimized indices of the elements are very close to the theoretical requirements. Non-dispersive elements have space-coiling geometry with local cavities, while dispersive elements contain solid blocks and complex cavities. Fortunately, the average transmission is guaranteed to be >80% during the optimization process (Fig. 4D). It has been found that only optimized asymmetrical topologies with multiple scatterers can realize the complex non-dispersive and dispersive ultra-broadband phase shift while maintaining a relatively high transmission. Figure 4E shows the generation of the beam at [16.5 kHz and 66 kHz], despite some shift in the ‘dark’ trapping regions. A small object can be suspended in mid-air by pushing and pulling forces [33], which are generated by the scattering of these dark areas. In the measurements, we used a polyethylene ball with a mass of 0.022 g and observed its stable single-sided suspension in air at representative frequencies (Fig. 4F and Supplementary Movies S1–S5). Limited by the single-frequency ability and fixed size of ultrasound transducers available in the market, we used mainstream products and conducted the levitation tests for 32 and 40 kHz. We also performed simulations by introducing thermo-viscous losses and confirmed that broadband performance is preserved (Supplementary Text S14). It is noted that most of the frequencies in Figs 4E, 4F and S24 are not directly considered in optimization. Therefore, it is noteworthy that the desired wave functionalities can be implemented across the entire bandwidth if an appropriate number of discrete sampling frequencies are selected (Supplementary Text S16).

### Mechanism of ultra-broadband achromatic dispersions

**Figure 5.**
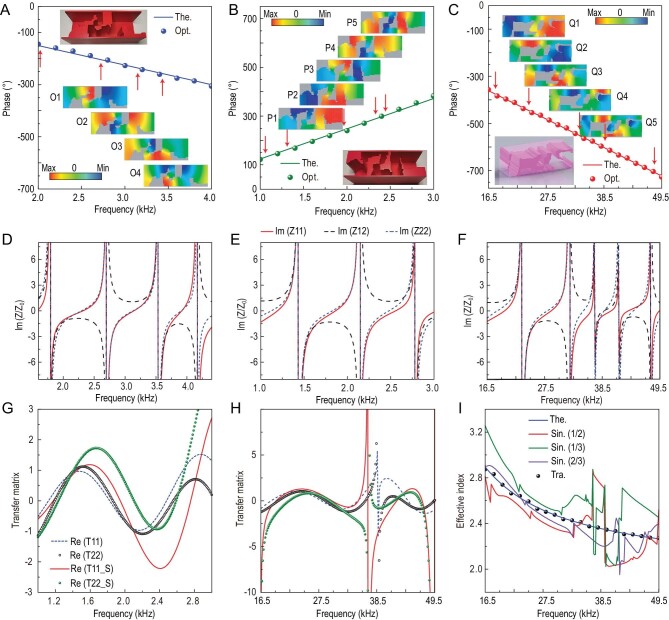
Ultra-broadband wave characterizations of typical optimized metasurface elements. (A)–(C) Theoretical (The.) and optimized (Opt.) phase-shift profiles of the three typical optimized elements for wave deflection (Element #6) (A), focusing (Element #7) (B) and levitation (Element #0) (C) engineering. The inserts display layouts and special acoustic pressure fields of the optimized elements. O1–O4, P1–P5 and Q1–Q5 represent the fields at frequencies indicated by the red arrows from left to right. These internal resonances collectively contribute to strong phase compensations at different frequencies. (D)–(F) Impedance matrix profiles of the optimized elements in A (D), B (E) and B (F). (G) and (H) Transfer matrix profiles of the elements in (A) and (C) without (T11 and T22) or with (T11_S and T22_S) the single-scattering assumption. (I) Comparison of theoretical (Theo.), traditional retrieved (Tra.) and single-scattering (Sin.) induced effective index of optimized Element #0 for the levitation engineering. Single-scattering results are derived by the model divided into half (1/2), one-third (1/3) and two-thirds (2/3) of Element #0.

To understand the physics behind the achieved ultra-broadband operations, we closely examine phase, effective impedance matrices and multiple-scattering effects for representative metasurface elements (Fig. 5). Regardless of beam deflection, focusing or levitation, the optimized asymmetrical space-coiling-cavity elements generate internal resonances in different regions of the structure at different frequencies (Fig. 5A–C), thus compensating for the complex phase shift resulting from the dispersion of individual components. Figure 5D–F shows that three representative elements also exhibit bi-anisotropy [20]. For a given functionality, the degree of bi-anisotropy of an element increases with its effective index or degree of dispersion (Supplementary Texts S9, S11 and S13). Consequently, more complex functionality usually requires stronger bi-anisotropy of the elements. Since bi-anisotropy is related to the structural symmetries, stronger bi-anisotropy can enhance multiple scattering and interaction of different individual components. We also find a topological feature that differs from the traditional metasurfaces using space-coiling [10,11] and Helmholtz-resonator [18,20] structures, namely multiple asymmetric solid blocks distributed in space. To evaluate the interaction between solid blocks, we obtain the transfer matrix profiles and effective indices by dividing the element into different sections (Supplementary Texts S6–S8) and analysing the scattering properties. In this way, multiple scattering is evaluated (Fig. 5G–I) and compared with the theoretical result. Regardless of how we divide the element, the characteristics of single-scattering are completely different from theoretical and obtained ones, which implies the presence of very strong multiple scattering, which plays a crucial role in determining the general characteristics of the metasurface. Broadband properties would not have been possible without this effect. In addition, both non-dispersive and dispersive elements exhibit similar multiple-scattering effects (Figs S25 and S26), although the latter generally require stronger multiple scattering. For both non-dispersive and dispersive properties, every optimized element displays entirely different multiple-scattering features (Figs 5G–I, S10, S11, S17, S18, S25 and S26). Hence, all metasurface elements exhibit diversified multiple scattering that is unlikely to be intuitively designed. On the other hand, topology optimization implicitly emphasizes the importance of the multiple scattering, which can be seen as a new and additional degree of the design. In general, all of the optimized elements can jointly support suitable integrated internal resonances, bi-anisotropy and multiple-scattering effects simultaneously to realize ultra-broadband functionality with high efficiency.

The space-coiling and Helmholtz-resonator structures do not naturally accommodate bi-anisotropy and multiple scattering. However, all three mechanisms are important in our analyses (resonance, bi-anisotropy and multiple scattering), which further leads to achromatic functionality. On the one hand, most Helmholtz-resonator and space-coiling structures can only produce high transmissions near resonant frequencies. But our elements provide relatively high transmission over a broadband frequency range. This is the essential difference between our elements and traditional resonant ones. On the other hand, most of the existing Helmholtz-resonator and space-coiling metasurfaces are limited by a certain dispersion property—that is, the characteristic of the wave functionality (for example, focal length, refraction angle, etc.) changes markedly with frequency. But our metasurfaces can support the non-dispersive nature of the wave functionality, which is another distinctive feature. Third, the effective metamaterial index of the space-coiling structure can be numerically characterized by the length of the propagation path. However, this approach fails to characterize the effective movements of our elements. In other words, the effective index is not directly related to the length of the propagation path. Therefore, we believe that the proposed elements differ from the previous ones using Helmholtz-resonator (HR) or coiling structures.

In short, we believe that the physical mechanisms of our inverse-designed elements are different from previous Helmholtz-resonator and space-coiling structures. Only the synergistic effect of internal resonances, bi-anisotropy and multiple scattering can simultaneously contribute to the ultra-broadband achromatic wave functionalities with a high transmission that has never been realized before. This discovery would provide a way to achieve ultra-broadband functionalities of metamaterials (though not yet attempted) through the combined effects of multiple mechanisms described above.

According to the acoustic intensity distributions (Supplementary Text S15), it is the amplification and complex path of the acoustic intensity flux that generates the ultra-broadband phase compensation and high transmission.

## CONCLUSION

By integrating phase modulation, transmission, geometric features and wave functionalities into the design, we have methodically demonstrated the deflection of an ultra-broadband acoustic beam and the focusing and levitation of achromatic metasurfaces following a bottom-up topology optimization. All optimized structures can represent the new category of AMs, which can support asymmetric multiple scatterers, curve air channels and local cavities simultaneously. We have found that the optimized asymmetric metasurfaces can appropriately combine integrated internal resonances, bi-anisotropy and multi-scattering effects simultaneously, resulting in ultra-broadband customized dispersions. This highly efficient ultra-broadband mechanism is expected to become a new and versatile benchmark for broadband AMs. In view of the robustness and versatility of the proposed metasurfaces, the reported ultra-broadband topological features and tunable dispersion mechanisms can provide a new impetus to the entire field of optical [34] (Supplementary Text S17), acoustic [35] and elastic metamaterials [36]. The broadband achromatic beam deflection can result in stable directional acoustic energy radiation and noise shielding. The broadband achromatic acoustic focusing can realize high-definition medical ultrasound images and high-intensity energy capture. Levitation of broadband achromatic ultrasound allows precise manipulation of particles of different weights and sizes.

Based on the intrinsic physical parameters, our constructed topology-optimization model enhances the existing design capability and the applicability of the AMs. The proposed design paradigm can be extended to other waves (optical, elastic) for their manipulation as well as the realization of more diversified functionalities such as absorption, acoustic insulation, vibration control and underwater acoustic manipulation, etc. Whenever a target functionality requires the tuning of specific dispersion, the proposed approach should be applicable to generate various broadband achromatic meta-devices. Meanwhile, the principle of stable single-sided ultrasound levitation can be extended to the area of acoustic tweezers. The dispersion-based wave manipulation method can also be generalized to vortex acoustics, holographic, 3D acoustic field control, etc.

A limitation of the proposed approach may be the complex geometry of inverse-designed structures. However, with the advent of advanced additive manufacturing capability, this could be mitigated in the foreseeable future. Limited by computational capacity, the current optimization process is applied to a 2D system. In terms of 3D design, dramatically increased design freedom will allow more complex wave functionalities with better performance and even wider bandwidth. Finally, combing our designs with existing active control and deep learning techniques will make large-scale, digital, reconfigurable and integrated achromatic metasurface devices available.

## METHODS

### Sample fabrication

Metasurfaces for deflection and focusing were manufactured using a UnionTech Lite800HD 3D-printing machine (Wenext, printing resolution: 200 μm) using a low-viscosity photosensitive resin with a mass density of 1250 kg/m^3^ and Young's modulus of 2370–2650 MPa. The metasurface for levitation was manufactured by a Micro Scale 3D Printing System nanoArch® S140 (BMF Material Technology Inc., printing resolution: 10 μm) using a low-viscosity high-temperature resistant resin with a mass density of 1150 kg/m^3^ and Young's modulus of 4.2 GPa.

## Supplementary Material

nwac030_Supplemental_Files
