# Supplementary material for: Achromatic metasurfaces by dispersion customization for ultra-broadband acoustic beam engineering
Source: Natl Sci Rev. 2022 Feb 24;9(12):nwac030. doi: 10.1093/nsr/nwac030 (PMC9883680; doi:10.1093/nsr/nwac030)
Supplement: nwac030_Supplemental_Files [file nwac030_supplemental_files.zip › SupplementaryMaterials-F.pdf]

Supplementary Materials for

Achromatic metasurfaces by dispersion customization for ultra-broadband acoustic beam engineering

Hao-Wen Dong (董浩文)†, Chen Shen (沈宸)†, Sheng-Dong Zhao (赵胜东)†, Weibao Qiu (邱维宝), Hairong Zheng (郑海荣), Chuanzeng Zhang (张传增), Steven A. Cummer, Yue-Sheng Wang (汪越胜)*, Daining Fang (方岱宁)*, Li Cheng (成利)*

***Corresponding authors.** E-mails: yswang@tju.edu.cn; fangdn@bit.edu.cn; li.cheng@polyu.edu.hk
†These authors contributed equally to this work.

This PDF file includes:

Supplementary Texts S1 to S21
Figs. S1 to S32
Captions for Movies S1 to S8

Other Supplementary Materials for this manuscript include the following:

Movies S1 to S8 (Online)

S1. Theory of designing ultra-broadband acoustic beam deflection

Inspired by the generalized Snell's law, we can systematically construct a specific transverse phase gradient of the metasurface to realize a desired refractive beam for airborne sound. When waves are incident on an artificial structure, the refraction angle can be related to the incident angle and phase gradient

$$\frac{1}{\lambda_t}\sin\theta_t - \frac{1}{\lambda_i}\sin\theta_i = \frac{1}{2\pi}\frac{d\phi}{dx}, \tag{1}$$

where λ_t and λ_i indicate the wavelengths of two medium; θ_t and θ_i are the angles of refraction and incidence, respectively; and $d\phi/dx$ is the gradient of phase discontinuity along the interface between two media. Equation (1) clearly implies that only the design considering the variation of phase shift with frequency can accomplish the truly broadband wave manipulation. In other words, the designed structure for broadband exotic refraction of airborne sound has to keep the term $(c_{air}/\omega)\times(d\phi/dx)$ constant over the wide frequency range, where ω denotes the angular frequency. Here we perform topology optimization of 6 elements to construct the metasurface for broadband beam deflection. The corresponding theoretical phase shift distributions for all elements are shown in Fig. S8. In addition, figure S13 depicts the theoretical simulations of beam deflection using the ideal phase shift distributions.

S2. Theory of designing ultra-broadband acoustic focusing

To focus the incident waves at a certain location away from the metasurface, the relative phase distribution provided by the metasuface should follow

$$\phi(x,\omega) = \frac{\omega}{c_{\text{air}}}\left(\sqrt{x^2+F_0^2} - F_0\right), \tag{2}$$

where x and F_0 are the coordinate and focal length, respectively. To implement the achromatic metasurface, we have to make the transverse wavevector of $d\phi(x,\omega)/dx$ constant with any different given coordinate and frequency. Unlike the reported approaches for the optical achromatic metalens [4], we use topology optimization to directly design every element with the desired phase shifts at every sampling frequency, explicitly tailor-making all phase performances on demand. The theoretical phase shift distributions for all 7 elements are shown in Fig. S15. In addition, figure S20 depicts the theoretical simulations of focusing using the ideal phase shift distributions.

S3. Theory of designing ultra-broadband acoustic levitation

To show the enormous potential of wavefront shaping, metasurfaces can be elaborately designed to generate the so-called bottle beam and then lead to the typical single-sided levitation [5-7]. However, neither the existing ultrasonic phase arrays nor the two-layer metamaterial bricks are able to provide ideal phase manipulation only by a single structure without sophisticated regulation and equipment, not to mention the stable levitation over a broadband frequency range. To realize the levitation near the focal region, the superimposed phase distribution provided by the metasurface can be expressed as

$$\begin{gathered}\phi(x,y,\omega) = \frac{\omega}{c_{\text{air}}}\left(\sqrt{x^2+y^2+F_0^2} - F_0\right) + \phi_0(x,y), \\ \phi_0(x,y) = \begin{cases} 0, & (\sqrt{x^2+y^2} \le r_0) \\ 180, & (\sqrt{x^2+y^2} > r_0) \end{cases},\end{gathered} \tag{3}$$

where x (y) and F_0 are the coordinate and the focal length created by the focusing decomposition field of the bottle beam, respectively; ϕ_0 is the holographic signature phase; and r_0 means the region whose phase is

opposite to the outer region. Apparently, a straightforward way to realize the broadband levitation is to make the spatial- and frequency-dependent transverse wavevector $d\phi(x, y, \omega)/dr$ constant for any location and frequency, where r denotes the spatial coordinate $\sqrt{x^2 + y^2}$. To tactically design this complex functionality, we also use topology optimization to explore the metasurfaces with 13×13 elements possessing the phase distribution extremely close to the theoretical one (Fig. S23). Finally, a 3D inversely designed metasurface will be assembled by the 2D optimized 28 elements. In addition, figure S27 depicts the theoretical simulations of levitation using the ideal phase shift distributions.

S4. Dispersions for three functionalities

To obtain the wave functionalities described by Eqs. (1)-(3), the metasurfaces must meet specific phase profile requirements at different incident wavelengths simultaneously. To understand this feature, Taylor expansion is applied to an arbitrary spatial- and frequency-dependent phase profile $\phi(r, \omega)$ as

$$\phi(r,\omega) = \phi(r,\omega_{\mathrm{d}}) + \left.\frac{\partial\phi(r,\omega)}{\partial\omega}\right|_{\omega_{\mathrm{d}}}(\omega-\omega_{\mathrm{d}}) + \left.\frac{\partial^2\phi(r,\omega)}{2\partial\omega^2}\right|_{\omega_{\mathrm{d}}}(\omega-\omega_{\mathrm{d}})^2 + O(\omega^3), \tag{4}$$

where ω_{d} denotes the design frequency; $\partial\phi(r, \omega)/\partial\omega$ and $\partial^2\phi(r, \omega)/\partial\omega^2$ are the relative group delays (GD) and relative group delay dispersions (GDD), respectively, which characterize the chromatic functionality shift of the metasurfaces. In another word, the GD compensates for the differences during the propagation of the wave packets, while the GDD ensures identical outgoing wave packets. Unfortunately, the traditional diffractive metasurfaces can only possess the required phase profiles at a given frequency, resulting in obvious dispersion and narrow-band properties. Achromatic functionality and the broadband feature can only be obtained when all terms in Eq. (4) are properly considered.

The GD of an achromatic metasurface for the beam deflection, focusing and levitation can be respectively defined by

$$\begin{cases} \dfrac{\partial\phi(x,\omega)}{\partial\omega} = \dfrac{x(\sin\theta_{\mathrm{t}} - \sin\theta_{\mathrm{i}})}{c_0}, \text{ for beam steering;} \\ \dfrac{\partial\phi(x,\omega)}{\partial\omega} = \dfrac{\sqrt{x^2+F_0^2}-F_0}{c_0}, \text{ for focusing;} \\ \dfrac{\partial\phi(r,\omega)}{\partial\omega} = \dfrac{\sqrt{r^2+F_0^2}-F_0}{c_0}, \text{ for levitaion.} \end{cases} \tag{5}$$

To achieve achromaticity, the essential physical characteristic quantities θ_t and F_0 in Eqs. (5) should be constant and frequency-independent, namely

$$\frac{\partial^2\phi}{\partial\omega^2} = 0. \tag{6}$$

To show the intrinsic dispersion of aforementioned metasurfaces, Fig. 1 depicts the effective indices for representative elements within a broadband range, and the required GDs and GDDs for all elements aiming at different functional characteristic quantities. As shown in Figs. S1(A)-1(C), the metasurface elements for achromatic beam deflection should be non-dispersive. The effective refractive index (Fokin et al., 2007) linearly increases with the element location. Similarly, the metasurface elements for achromatic focusing are non-dispersive as well. However, the change of element location results in the nonlinear increase of the effective index. Moreover, achromatic levitation requires more complex distribution of the effective index. Part of elements has the constant index within the broadband range, whilst others showing strong dispersions, i.e., the effective index decreases with increasing frequency. In addition to the effective index, the rigorous GDs and GDDs are also needed. Three functionalities show different group delays and group delay

dispersions in Figs. S1(D)-S1(F). In another word, each element has to possess a different group delay. But all elements have zero group delay dispersion for the achromatic functionality. For beam deflection, the group delay increases with the distance from the center location. A larger refractive angle implies a larger group delay for the element at the same location. For both focusing and levitation, the group delay nonlinearly increases with the distance from the center location. When the focusing or levitation position is sufficiently small, the group delay profile becomes nearly linear. As illustrated in Figs. S1(E) and S1(F), a larger focusing or levitation position usually means stronger nonlinear feature. The curves with very small positions become nearly linear. Actually, these specific requirements can not only guide the realization of achromatic functionalities but also allows the prediction of their limits.

In summary, in order to realize an achromatic design, all elements at different locations of a metasurface have to satisfy specific effective refractive indices, relative group delays and relative group delay dispersions simultaneously.

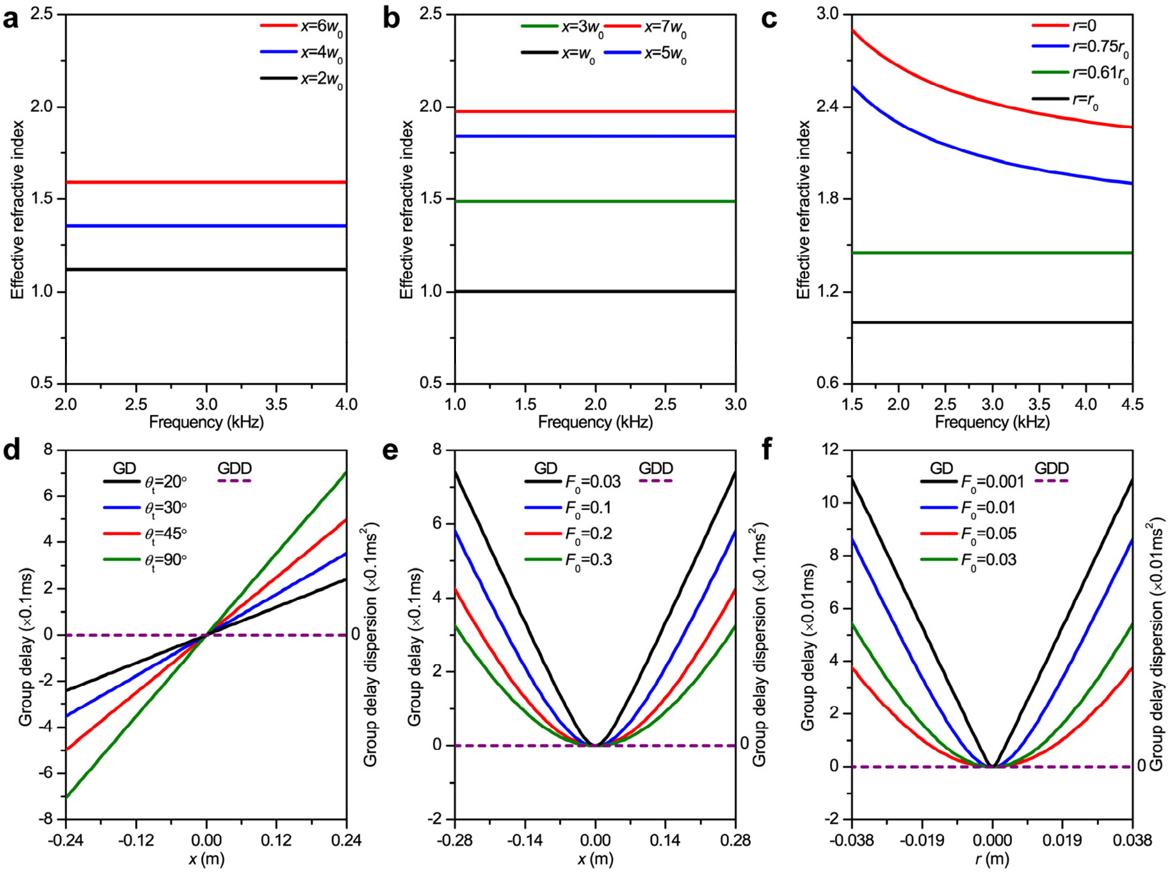

Fig. S1. Dispersion engineering of achromatic metasurfaces. (a-c) Effective indices of representative metasurface elements for beam deflection (a), focusing (b) and levitation (c). (d-f) Required relative group delays (GD) and relative group delay dispersions (GDD) as a function of metasurface coordinate for deflection (d), focusing (e) and levitation (f). Solid and dash lines express the relative GD and GDD, respectively.

S5. Setup and procedure of topology optimization

To construct a metasurface with the desired functionality, we use topology optimization to systematically design metasurface elements with the required phase distributions at different frequencies. In the meantime, we have to introduce special physical restrictions to ensure high transmission. During optimization, an element can include several closed regions, which leads to obvious narrow-band resonances. In addition, an optimized element usually has poor transmission phase control capability if it cannot remain connected

through the two ports of the design domain. Moreover, an optimized element cannot be efficiently manufactured if its minimal solid component is too thin to ensure sufficient strength and manufacturing compatibility. It is likely that very narrow air channels will also lead to noticeable viscosity dissipation. Therefore, we also need to introduce some geometric constraints to make the optimized elements manufacturable and physically sane. For any desired broadband functionality, the optimization problem is formulated as

$$For: f_i \in [f_{\min}, f_{\max}]\,(i = 1, 2 \cdots N_{\mathrm{F}}), \tag{7}$$

$$Maximize: O(\Omega_{\mathrm{D}}) = \frac{-max\left\{\sqrt{\frac{\sum_{i=1}^{N_{\mathrm{F}}}(\phi_{\mathrm{D}}^i)}{N_{\mathrm{F}}}} \times \underset{\forall i \subset (1,2\cdots,N_{\mathrm{F}})}{max}(\phi_{\mathrm{D}}^i), \sqrt{\left[\frac{1}{N_{\mathrm{F}}}\sum_{i=1}^{N_{\mathrm{F}}}\left(\phi_{\mathrm{D}}^i - \frac{\sum_{i=1}^{N_{\mathrm{F}}}(\phi_{\mathrm{D}}^i)}{N_{\mathrm{F}}}\right)^2\right]} \times \underset{\forall i \subset (1,2\cdots,N_{\mathrm{F}})}{max}(\phi_{\mathrm{D}}^i)\right\}}{360}, \tag{8}$$

$$\begin{aligned} Subject\ to: &\ \rho_i = 0\ or\ 1\,(i = 1, 2 \cdots N_{\mathrm{EX}} \times N_{\mathrm{EY}}), \\ &\ \Omega_{\mathrm{A}} \geq 1, \\ &\ \underset{\Omega_{\mathrm{D}}}{min}(\boldsymbol{w}_{\mathrm{A}}) \geq w_{\mathrm{A0}}, \\ &\ \underset{\Omega_{\mathrm{D}}}{min}(\boldsymbol{w}_{\mathrm{S}}) \geq w_{\mathrm{S0}}, \\ &\ T(\Omega_{\mathrm{D}}) \geq 0.6, \end{aligned} \tag{9}$$

where f_i denotes the sampling frequency of the target frequency range [$f_{\min}$, $f_{\max}$], which is evenly divided into ($N_{\mathrm{F}}-1$) frequency subintervals by N_{F} discrete frequencies; Ω_{D} is the topological distribution in 2D design domain (Supplementary Sec. S1); O represents the objective function value for characterizing the degree of violation of theoretical phase shifts at all sampling frequencies; ϕ_{D}^i means the absolute difference between the desired phase shift and the theoretical at the sampling frequency; ρ_i indicates the density of the material of an element that can declare its airy (0) or solid (1) attribute; N_{EX} and N_{EY} are the numbers of finite elements of the design domain along the x and y directions; Ω_{A} expresses the number of connected air domains containing at least one connected area spanning two ports of the design domain; $\boldsymbol{w}_{\mathrm{A}}$ and $\boldsymbol{w}_{\mathrm{S}}$ denote arrays containing the sizes of all local components of air and solid, respectively; w_{A0} and w_{S0} are empirical values suggested by many optimization tests; T represents the minimal value (beam deflection) or the averaged value (focusing and levitation) of the transmission coefficients at N_{F} sampling frequencies. A constraint value of 0.6 has been suggested by several optimization tests.

To systemically design the metasurface elements on demand, we utilize topology optimization based on discrete variables to explore near-optimal elements. Though we do not intent to claim any novelty in the optimization algorithm, we still provide brief description of the procedure for the sake of completeness of the paper. As displayed in Fig. S2a, a design domain Ω_{D} of $W \times H$ is meshed by $N_{\mathrm{EX}} \times N_{\mathrm{EY}}$ 4-node linear acoustic quadrilateral finite elements (AC2D4) in ABAQUS 6.14-1. In fact, the air regions Ω_{L} and Ω_{R} can be taken as the lattice structure composed of the periodic air microstructures. Therefore, the periodic boundary conditions are introduced in order to consider the effects of adjacent air region. Otherwise, the transmitted phase and transmission of the microstructure retrieved by the model in Fig. S2a cannot be coincident with the assembled metasurface. Note that we forcibly add two solid parts on the top and the bottom of the design domain to avoid the coupling between optimized microstructures. With incoming incident plane waves at y_6, the classic four-microphone method [1] is employed to compute the transmission coefficient. Four microphones used for measuring acoustic pressures are placed at y_1, y_2, y_3 and y_4. The absolute transmission phase of the microstructure is extracted from the response at y_5. After measuring the acoustic pressures at y_1, y_2, y_3 and y_4,

the acoustic pressures and particle velocities at y_0 and y_d are obtained and marked by p_0, p_d, v_0 and v_d, respectively.

Since we just focuse on the forward transmission (i.e., waves propagate from the left to right direction in Fig. S2a), the transfer matrix **T** of the effective two-port network can be written as

$$\mathbf{T}=\begin{bmatrix} T_{11} & T_{12} \\ T_{21} & T_{22} \end{bmatrix}=\begin{bmatrix} \dfrac{p_d v_d + p_0 v_0}{p_0 v_d + p_d v_0} & \dfrac{p_0^2 - p_d^2}{p_0 v_d + p_d v_0} \\ \dfrac{v_0^2 - v_d^2}{p_0 v_d + p_d v_0} & \dfrac{p_d v_d + p_0 v_0}{p_0 v_d + p_d v_0} \end{bmatrix}. \tag{10}$$

Due to the transformation relationship between the scattering and transfer matrices [1], the scattering matrix **S** can be generated as

$$\mathbf{S}=\begin{bmatrix} S_{11} & S_{12} \\ S_{21} & S_{22} \end{bmatrix}=\begin{bmatrix} \dfrac{T_{11}+T_{12}/Z_0-T_{21}Z_0-T_{22}}{T_{11}+T_{12}/Z_0+T_{21}Z_0+T_{22}} & \dfrac{2(-T_{21}T_{12}+T_{11}T_{22})}{T_{11}+T_{12}/Z_0+T_{21}Z_0+T_{22}} \\ \dfrac{2}{T_{11}+T_{12}/Z_0+T_{21}Z_0+T_{22}} & \dfrac{-T_{11}+T_{12}/Z_0-T_{21}Z_0+T_{22}}{T_{11}+T_{12}/Z_0+T_{21}Z_0+T_{22}} \end{bmatrix}, \tag{11}$$

where S_{11} and S_{21} are the reflection and transmission coefficients R and T, respectively. The transmission used in all results in the body text and supplementary materials of the paper is defined as the modulus of transmission coefficient T, i.e., $|T|$ [1, 2].

Different thicknesses of the top/bottom thin solid plate h_0 are selected for the beam deflection (h_0=H/80), focusing (h_0=H/80) and levitation (h_0=H/40). In the optimizations of the beam deflection and focusing, H and W take the values of 4 mm and 1.2 cm, respectively. In view of the ultrasonic realizations, the values of H and W for the levitation are defined as 0.4 mm and 1.2 mm, respectively.

To realize the wave functionality on demand, one can define the corresponding dispersion of every component element and the dispersion of the whole metasurface macrostructure. Subsequently, one can deduce the phase shift of every element at every discrete sampling frequency and define a minimal averaged transmission limit of the element within the target frequency range. These two kinds of physical quantities will be employed to evaluate the fitness of every individual in genetic algorithm (GA). With suitable coding and decoding process, all metasurface elements involved in the optimization can be treated as individuals (chromosome) to evolve into the best one after proper selection, crossover and mutation processes. Each individual is composed of $2^{N_{EX}\times N_{EY}}$ design variables attached to a binary matrix. In the initial stage of GA, a random population including N_p binary individuals is generated. This initial population also represents the first generation of parent population. To eliminate the checkerboard pattern, an "abuttal entropy filter" is adopted to remove some isolated pixels (finite elements) of 1 and fill up some isolated pixels of 0, thus improving the topologies and strengths of individuals. Then the fitness values and the constraint satisfactions of individuals are evaluated based on the objective function and constraints, i.e., the match degree ϕ_D of the exact phase performance with the target phase distribution, the averaged transmission T at all discrete sampling frequencies, the connectivity of the air regions Ω_A within the design domain, the minimal air $min(\boldsymbol{w}_A)$ and solid $min(\boldsymbol{w}_S)$ feature sizes, respectively. To breed a new generation, all individuals of a current population should be selected through the tournament selection scheme: i) randomly selecting N_{ts} individuals to create a competition group; ii) forcing all N_{ts} individuals to compete with each other; iii) putting the best individual into the mating pool; and iv) executing N_p competitions to retain the excellent N_p individuals for the next genetic operations. During the tournament selection, the competition follows the following rule: whenever possible, the individual i with a better fitness value will be selected; if neither individual is a feasible solution, the one with less violation of the constraints will be selected. Particularly, if two solutions have the same performances of the objective function and constraints, the solution with the fewer scatters (solid blocks) should be the better one. After selection (reproduction), the uniform crossover operation with crossover possibility P_c will be applied to the mating pool by exchanging partial genes of two individuals

according to a random binary mask. To allow for new possibilities of search space, the mutation operation with the mutate possibility P_m will be applied to the current generation subsequently. After a set of genetic operations, the algorithm generates an offspring generation. To accelerate the evolution, the elitism strategy is used at the end of each generation by copying the best individual from the current generation into the next generation. Hence, the new population is created and becomes the parent population for the next round of generation. If the predefined generation number E_G is reached, the procedure will stop and return an optimized metasurface element. Once all required elements are generated, one can assemble them into an inversely designed metasruface according to the specific configuration pattern determined by the desired functionality. Then one can calculate its exact dispersion property and check its exact functionality. Finally, the inversely designed metasuface will be manufactured into a 3D-printed sample for the experimental verifications. The detail procedure of whole inverse-design strategy is shown in Fig. S2b.

In theory, the topology optimization of metasurfaces based on the specific phase and transmission properties is a classic non-convex, multiple-constraint, massive-variable optimization problem. For a given phase ϕ, different structures with ($\phi \pm n \times 360°$, n is an integer) should deliver the same phase performance. Therefore, the optimization problem should have multiple-solutions, inferring that there should exist many microstructures with the same phase and transmission properties. In view of involving several strict geometrical constraints, our optimization model usually generates the similar asymmetric topologies after many runs. Note that the analysis on the effect of structural symmetry clearly shows that the asymmetrical topology of metasurface elements is the most beneficial feature for achieving perfect phase distributions and high transmissions. The material parameters in optimization are chosen as: ρ_{air}=1.29 kg/m^3, c_{air}=340 m/s, ρ_{solid}=1230 kg/m^3 and c_{solid}=2230 m/s. Because the impedance of solids is much larger than that of the air, generally, the solids can be assumed to be acoustically hard to simplify the optimization. The target frequency ranges in optimization are predefined as [2000 Hz, 4000 Hz], [1000 Hz, 3000 Hz] and [16.5 kHz, 49.5 kHz] for the beam deflection, focusing and levitation, respectively. In view of the different dispersive properties, the levitation engineering has to adopt more sampling frequencies (N_F=21) to capture the accurate phase shifts than those in the beam deflection (N_F=11) and focusing (N_F=11) engineering. To obtain structures with smooth edges in reasonable computing time, a "coarse to fine" two-stage strategy is applied in all optimizations. In the coarse stage, the design domain is meshed into 20×60 (N_{EX}=60, N_{EY}=20) pixels. In the fine stage, however, all optimized elements in the coarse stage serve as the initial population of new run of GA in the finer 40×120 (N_{EX}=120, N_{EY}=40) pixels. The parameters of minimal size constraints are set to w_{A0}=2mm, w_{S0}= 1mm for the beam deflection engineering, w_{A0}=2mm, w_{S0}= 1mm for the focusing engineering, and w_{A0}=200 μm, w_{S0}= 100 μm for the levitation engineering. The algorithm parameters of GA are the population size N_p=30, the crossover possibility P_c=0.9, the mutate possibility P_m=0.03, and the tournament competition group N_{ts}=21. To guarantee the near-optimality, every optimized element is selected from 5 optimizations with the same parameters. Numerous numerical tests show that sufficient generations can make the optimization converge to the ideal phase requirements. Specifically, the maximal generation number E_G takes 10000 (coarse) and 10000 (fine) for the beam deflection engineering, 3000 (coarse) and 10000 (fine) for the focusing engineering, and 2500 (coarse) and 5000 (fine) for the levitation engineering, respectively. It is worth noting that a more complex functionality contrarily demands fewer generations, implying easier capture of the evolution direction for a more complex phase requirement. All optimizations are conducted on a Linux cluster with Intel Xeon Platinum 8168 @ 2.70 GHz. The total time for an element optimization is about 93.3, 64.2 and 36.4 hours for the beam deflection, focusing and levitation engineering, respectively. The performances of the phase shift and transmission coefficient in optimization are computed by the commercial finite element software ABAQUS 6.14-1.

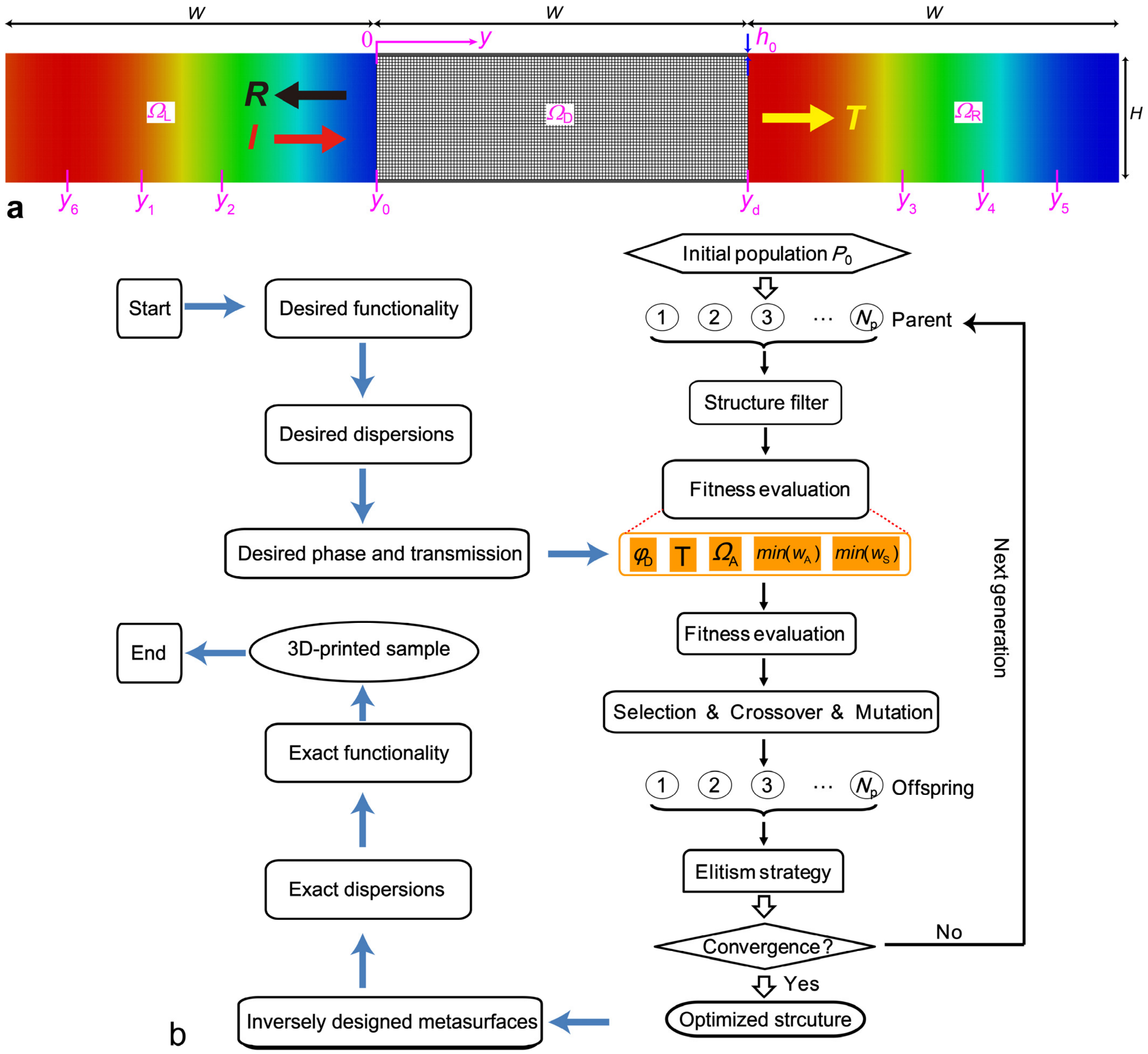

Fig. S2. Setup of the computational model and flow chart of the topology optimization. a, The white region indicates the design domain Ω_D without any symmetry assumption. The left and right air regions are marked by Ω_L and Ω_R, respectively. Periodic boundary conditions are imposed on the top and bottom edges of regions Ω_L and Ω_R. The simulation model is terminated with left and right nonreflecting boundaries. The positions of four microphones are y_1, y_2, y_3 and y_4, respectively. Two gray areas on the top and bottom of the design domain are artificially taken as solid parts to avoid the coupling between two adjacent optimized elements of the metasurface. b, Flow chart of the inverse design using GA.

S6. Impedance matrix of a bi-anisotropic metasurface element

In recent years, bi-anisotropic metasurfaces have been shown to provide a new way for wave manipulations by realizing the independent control of the reflection and transmission phases or the difference in the reflection phases [8-10]. In theory, asymmetrical element leads to the bi-anisotropic response at macroscale. In this case, the phase of reflection for both the forward and backward propagations is different

(asymmetrical), thus giving rise to the different scattering patterns depending on the direction of illumination. Since all optimized elements in our work are asymmetric, it is necessary to check the impedance matrices to evaluate their bi-anisotropic property. Using the four-microphone model in Fig. S2, we compute the responses in two cases. One case considers the nonreflecting boundaries at the right end of the model (#1), while, the other one adopts a hard wall at the right end (#2). The extracted pressure is expressed as P_i^j, where i denotes the sequence number of the microphone and j represents the case number. The complex sound pressures at four probing positions for the two cases are respectively expressed as

$$\begin{bmatrix} -e^{-ik_0y_1} & e^{-ik_0y_1} \\ -e^{-ik_0y_2} & e^{-ik_0y_2} \end{bmatrix}\begin{bmatrix} A^1 & A^2 \\ B^1 & B^2 \end{bmatrix}=\begin{bmatrix} P_1^1 & P_1^2 \\ P_2^1 & P_2^2 \end{bmatrix}, \tag{12}$$

and

$$\begin{bmatrix} -e^{-ik_0y_3} & e^{-ik_0y_3} \\ -e^{-ik_0y_4} & e^{-ik_0y_4} \end{bmatrix}\begin{bmatrix} C^1 & C^2 \\ D^1 & D^2 \end{bmatrix}=\begin{bmatrix} P_3^1 & P_3^2 \\ P_4^1 & P_4^2 \end{bmatrix}, \tag{13}$$

where k_0 is the wave number.

With the metasurface element located at y_0, the pressures and velocities on the left (−) and right (+) sides of the element are respectively determined by

$$\begin{bmatrix} p^{-1} & p^{-2} \\ v^{-1} & v^{-2} \end{bmatrix}=\begin{bmatrix} e^{-ik_0y_0} & e^{ik_0y_0} \\ e^{-ik_0y_0}/Z_0 & -e^{ik_0y_0}/Z_0 \end{bmatrix}\begin{bmatrix} A^1 & A^2 \\ B^1 & B^2 \end{bmatrix}, \tag{14}$$

and

$$\begin{bmatrix} p^{+1} & p^{+2} \\ v^{+1} & v^{+2} \end{bmatrix}=\begin{bmatrix} e^{-ik_0(y_0+d)} & e^{ik_0(y_0+d)} \\ e^{-ik_0(y_0+d)}/Z_0 & -e^{ik_0(y_0+d)}/Z_0 \end{bmatrix}\begin{bmatrix} C^1 & C^2 \\ D^1 & D^2 \end{bmatrix}, \tag{15}$$

where p, v, d and Z_0 are the pressure, the particle velocity, the width of metasurface element and impedance of the air, respectively.

Accordingly, the transfer matrix **T** relating the pressures and velocities on two sides becomes

$$\begin{bmatrix} T_{11} & T_{12} \\ T_{21} & T_{22} \end{bmatrix}=\begin{bmatrix} p^{+1} & p^{+2} \\ v^{+1} & v^{+2} \end{bmatrix}\begin{bmatrix} p^{-1} & p^{-2} \\ v^{-1} & v^{-2} \end{bmatrix}^{-1}. \tag{16}$$

Therefore, the impedance matrix **Z** can be calculated by

$$\begin{bmatrix} Z_{11} & Z_{12} \\ Z_{21} & Z_{22} \end{bmatrix}=\begin{bmatrix} -\dfrac{T_{22}}{T_{21}} & -\dfrac{1}{T_{21}} \\ \dfrac{T_{12}T_{21}-T_{11}T_{22}}{T_{21}} & -\dfrac{T_{11}}{T_{21}} \end{bmatrix}. \tag{17}$$

Furthermore, the scattering matrix **S** can be obtained by

$$\begin{bmatrix} r^{+} & t^{-} \\ t^{+} & r^{-} \end{bmatrix}=\begin{bmatrix} \dfrac{(Z_{11}-Z_0)(Z_{22}+Z_0)-Z_{21}Z_{12}}{(Z_{11}+Z_0)(Z_{22}+Z_0)-Z_{21}Z_{12}} & \dfrac{2Z_{12}Z_0}{(Z_{11}+Z_0)(Z_{22}+Z_0)-Z_{21}Z_{12}} \\ \dfrac{2Z_{21}Z_0}{(Z_{11}+Z_0)(Z_{22}+Z_0)-Z_{21}Z_{12}} & \dfrac{(Z_{11}+Z_0)(Z_{22}-Z_0)-Z_{21}Z_{12}}{(Z_{11}+Z_0)(Z_{22}+Z_0)-Z_{21}Z_{12}} \end{bmatrix}, \tag{18}$$

where t^+ and t^- mean the forward and backward transmission coefficients; and r^+ and r^- are the forward and backward reflection coefficients, respectively.

S7. Multiple-scattering model and effect characterization

If an element has many scatters, it is necessary to study its scattering property, either in the short or long wavelength scales. When an element is divided into m substructures, we can compute the corresponding

independent transfer matrix of every substructure using the model shown in Fig. S2a. In principle, the effective transfer matrix, $\mathbf{T}_S=\mathbf{T}_m\cdot\mathbf{T}_{m-1}\cdots\mathbf{T}_2\cdot\mathbf{T}_1$ for an assembly of m substructures in Fig. S3b, should be equal to that derived from the original model in Fig. S3a, if only single scattering exists. Certainly, in this case the values of T, R and I are the same as T_S, R_S and I_S. For the asymmetrical element, the same characteristic will be kept as well. Conversely, the two kinds of transfer matrices will be different if the multiple scattering is involved. As a result, the two kinds of effective indices and scattering matrices are different.

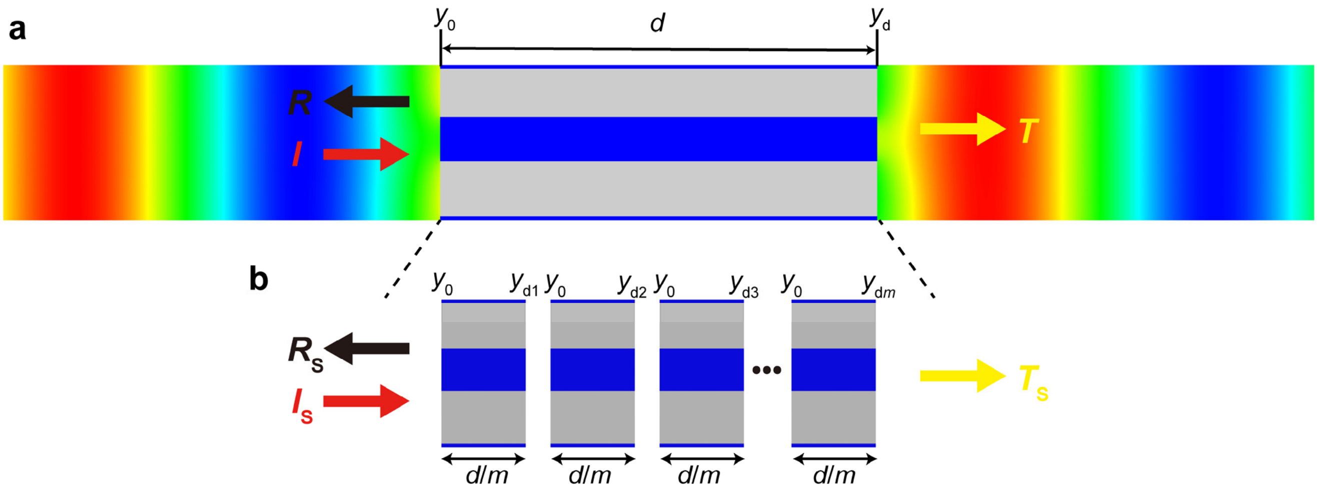

Fig. S3. A schematic element divided into m substructures. a, The model of a symmetrical element with the certain transmission and reflection under the incidence of acoustic waves. b, A set of substructures with the same size. Their effective transmission, reflection and incidence are marked by T_S, R_S and I_S, respectively. The blue and grey parts denote the solid and air elements, respectively.

To characterize multiple scattering of an optimized element, we divide the optimized element #0 for levitation engineering into two parts at three different locations (1/2, 1/3 and 2/3), see Fig. S4. For the multiple scattering factors, all optimized elements are conformably divided into two equal parts at the location 1/2.

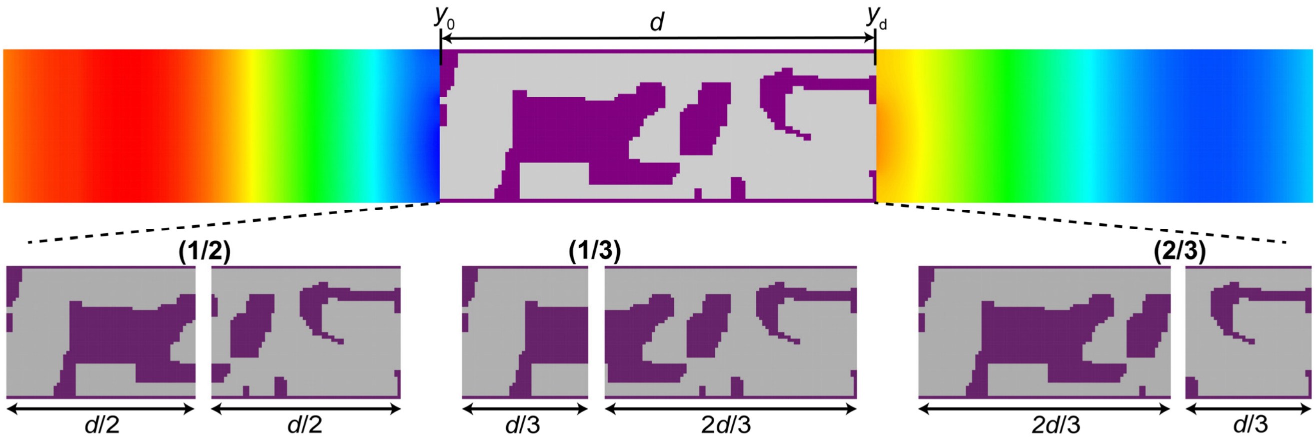

Fig. S4. A representative optimized element divided into two parts at different locations. The purple and grey parts represent the solid and air elements, respectively.

S8. Multiple-scattering factor of an optimized element

In the field of metasurfaces, the use of multiple scattering effects to modulate the dispersions and effective index has never been considered because of the complexity and harsh demand in the microstructural design. Since most elements in this paper have many distributed solid blocks, it becomes extremely complicated to regard every block as an independent scatterer. Here we adopt two steps to analyze the variation of the effective index to investigate the existence of multiple scattering and characterize the multiple-scattering effects of the optimized elements. In the first step, the traditional inverse technique [11] is utilized to retrieve the corresponding transfer matrix $\mathbf{T_O}$, scattering matrix $\mathbf{S_O}$ and effective index n_O of an optimized element. In the second step, the optimized element is divided into several substructures (Supplementary text S7) whose respective transfer matrix $\mathbf{T}_i$ (i=1, 2···m) is calculated respectively, where m is the total number of substructures. Then we can get the effective transfer matrix $\mathbf{T_S}=\mathbf{T}_m\cdot\mathbf{T}_{m-1}\cdots\mathbf{T}_2\cdot\mathbf{T}_1$ of the element based on the single scattering assumption. As a result, the effective single-scattering index n_S will be induced by the effective single-scattering transfer matrix $\mathbf{T_S}$. To characterize the multiple-scattering extent of the element, we define the multiple-scattering factor χ as

$$\chi = 2\frac{|n_O - n_S|}{n_O + n_S}, \tag{19}$$

where both n_O and n_S are positive for all optimized elements within the target frequency range. Note that χ becomes zero for the traditional space-coiling and Helmholtz-resonator metasurfaces [12]. However, the obvious multiple scattering effects exist in our optimized elements.

S9. Characterizations of optimized elements for ultra-broadband beam deflection

To obtain the desired metasurfaces with customized dispersion, we systematically study the topology optimization of a series of metasurface elements to construct a rigorously manipulated wavefront. For all optimization results presented in this paper, we run the optimization algorithms at least 6 times and then select the best optimized solution as the final optimized element for any phase engineering case. During these 6 optimizations, one part of them adopts randomly generated structure as the initial “seed” design, whilst the other part introduces the air structure in the initial evolution.

For ultra-broadband beam deflection, except Element #1, we perform topology optimization of every element according to the required phase shift relative to its previous element. This approach aims at reducing the search space of the optimization to speed up convergence. As illustrated in Fig. S5 (Supplementary Movies S6-S8), the evolutions of optimized Elements #4, #5 and #2 clearly show their forming processes. Although 20000 generations are predefined in the optimization, the algorithm quickly captures the beneficial topological features for Elements #4, #5 and #2 at generations G=324, G=646 and G=111, respectively. Compared with Elements #4 and #5, the evolution of Element #2 undergoes the fastest convergence, implying that the element with smaller index is easier to be designed. As shown in Fig. S5d, all optimized elements can realize the ideal fitness, while commendably keeping the high transmission property. This validates the effectiveness of the proposed design methodology in engineering metasurfaces for ultra-broadband beam deflection.

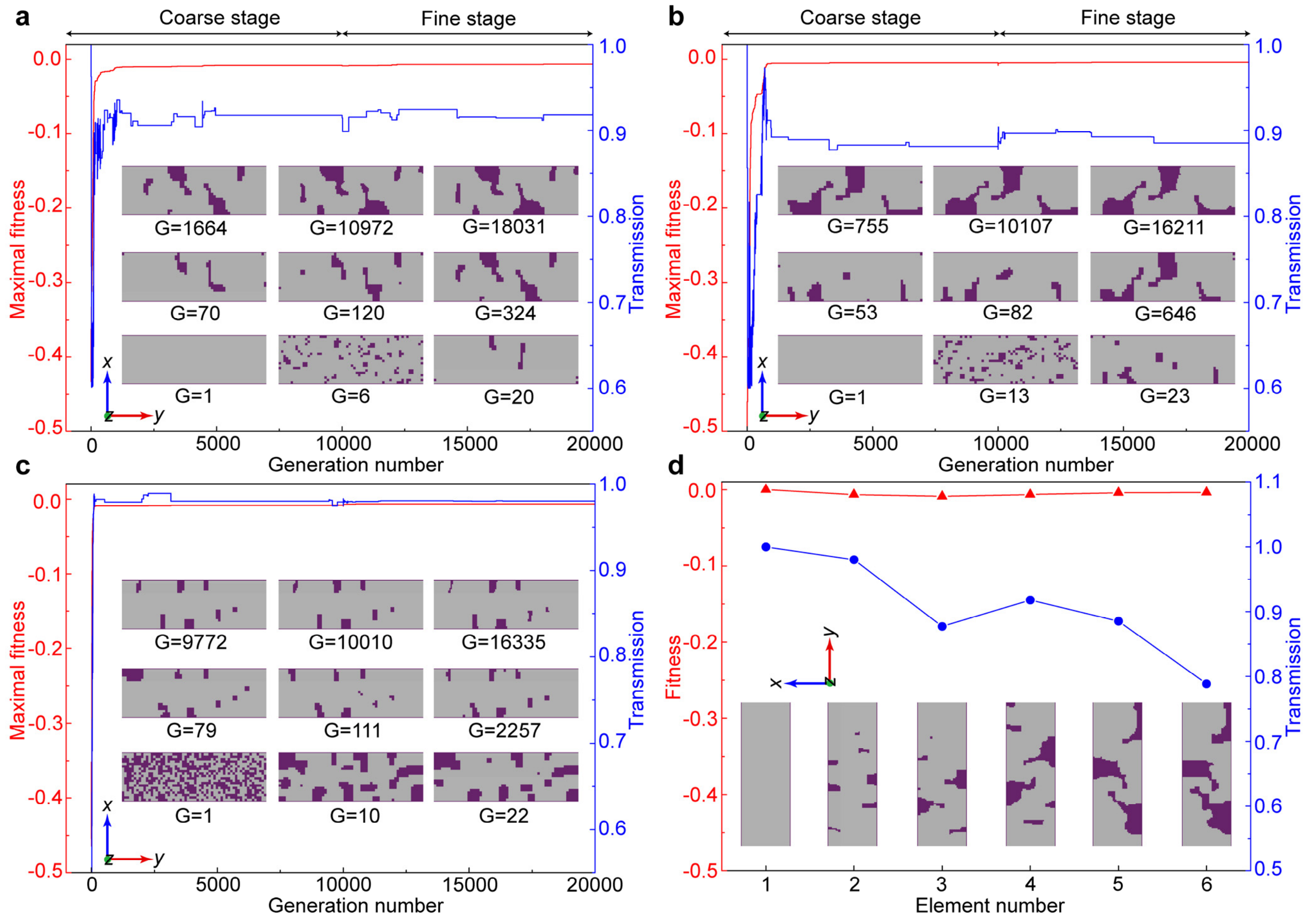

Fig. S5. Optimization performance of representative optimized elements in Fig. 2. a-c, Evolution histories of Elements #4 (a), #5 (b) and #2 (c) in Fig. 2, respectively. The first and last 10000 generations show the evolutions at the coarse and fine stages, respectively. Subgraphs display the representative topologies during evolution. d, Fitness and transmission values of all optimized elements. The purple and grey parts represent the solid and air elements, respectively.

Given the significance of the structural symmetry in topology optimization, it is essential to ensure the best symmetry for the ideal fitness and transmission. As presented in Fig. S6, we investigate different optimization results under four typical structural symmetries, i.e., the asymmetry, orthogonal, horizontal and vertical symmetries. With the same initial randomly generated structure, four symmetries generate four kinds of metasurface elements, see Fig. S6a. In particular, the optimized vertically symmetric element is significantly worse than the other three cases. We design an improved vertically symmetric element (vertical symmetry-2) after changing the initial design as the air structure. Five evolution and transmission curves clearly demonstrate that the asymmetry is the best geometrical assumption which possesses the maximal fitness and satisfactory high transmission. Additionally, the optimized asymmetric element has the fewest solid blocks, markedly reducing the manufacturing difficulty. In fact, the orthogonal and horizontal symmetries are also very effective because of their relatively good fitness and nearly 100% transmission. On one hand, the phase distribution is the foremost quantity in topology optimization, prior to transmission. One the other hand, the asymmetry has the largest search space for the same design domain Ω_D. Therefore, we select the asymmetry assumption for all optimizations in the present paper, including the beam deflection, focusing and levitation.

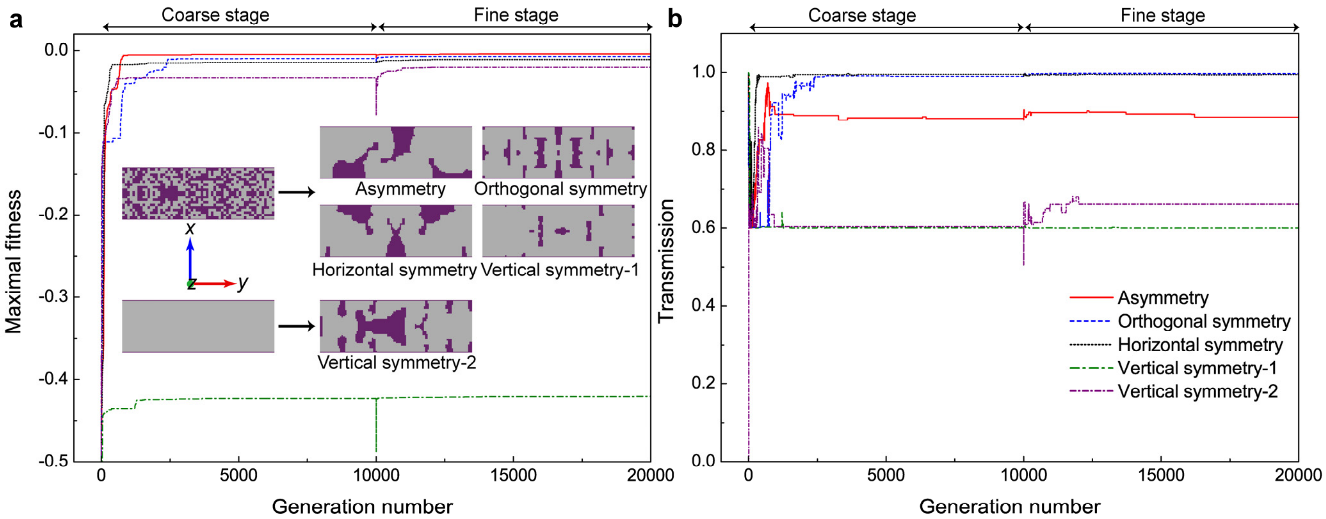

Fig. S6. Importance of the asymmetry to optimization. a-b, Evolutions of the fitness (a) and transmission (b) under the different representative symmetries for the generation of Element #5 in Fig. 2. The purple and grey parts represent the solid and air elements, respectively.

As shown in Fig. S7, the optimized asymmetric elements exhibit common topological features: 1) several discrete asymmetric solid blocks; 2) several local cavities; and 3) curved air channels. The air channel of the optimized element becomes narrower when a larger index is needed. Apparently, this feature conversely confirms the necessity of the minimal geometry constraints in the proposed optimization strategy. Here it remains to be explained that the air structure #1 is adopted according to its ideal constant index at any frequency. In other words, the air structure is essentially ultra-broadband element. Hence, comparing the subsequent element with the air structure should be the best choice for realizing the ultra-broadband metasurfaces. This design approach is also introduced in the following optimizations for focusing and levitation.

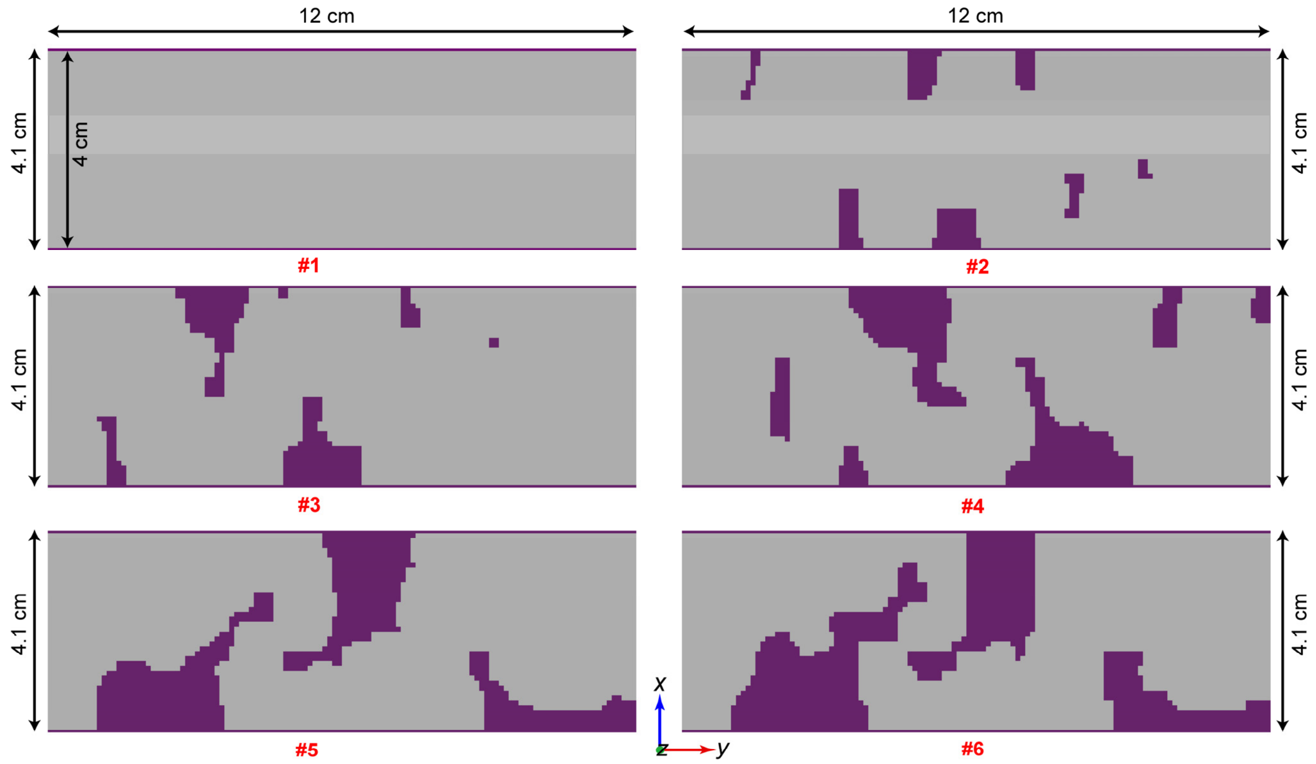

Fig. S7. Topologies of all optimized elements for ultra-broadband beam deflection. The purple and grey parts represent the solid and air elements, respectively.

As illustrated in Fig. S8a, the phase shifts at the sampling frequencies are in good agreement with the theoretically required ones. Overall, there exit small differences between the two kinds of the phase distributions. In theory, the optimized phase distribution will be better if the transmission requirement can be relaxed. In view of the specific topologies of optimized elements in Fig. S6, the whole transmission will decrease in varying degrees with the increase of effective indexes. Figure S8b shows the transmission spectra and the wave responses with the minimal transmission coefficients for four representative elements. Since Element #1 nearly equals to an "air" structure, its wave response R1 features almost 100% transmission within the entire frequency range. This means that the thin solid walls on the top and the bottom edges of region Ω_D do not affect the transmission property of the element. On account of the asymmetric solid blocks distributed in Ω_D, however, the typical internal resonances appear because of the local cavities. At the same time, part of the energy is reflected by the solid scatters and then causes the decreased transmission.

To further check whether the Fabry-Perot (FP) resonance is induced in this case, we compare the theoretically calculated FP resonance frequency with the exact frequency corresponding to the transmission valley of Element #6. According to the typical Fabry-Perot (FP) resonance condition $kd=N\pi$, we can subsequently derive the FP resonance frequency by $f_{FP}=(c_0N)/(2n_{eff}d)$, where c_0 stands for the sound velocity of the air; n_{eff} is the effective index of the microstructure; N is an integer; and d is the thickness of the element. The theoretically calculated f_{FP} for Element #6 yields 4454 Hz which is about 5% larger than 4246 Hz with 78.6% transmission in Fig. S7b. This means that the slight transmission reduction of Element #6 is not caused by the FP resonance effect, but the internal resonance. Similarly, we have also checked this issue for the other five elements and found no evidences of the FP resonance effects.

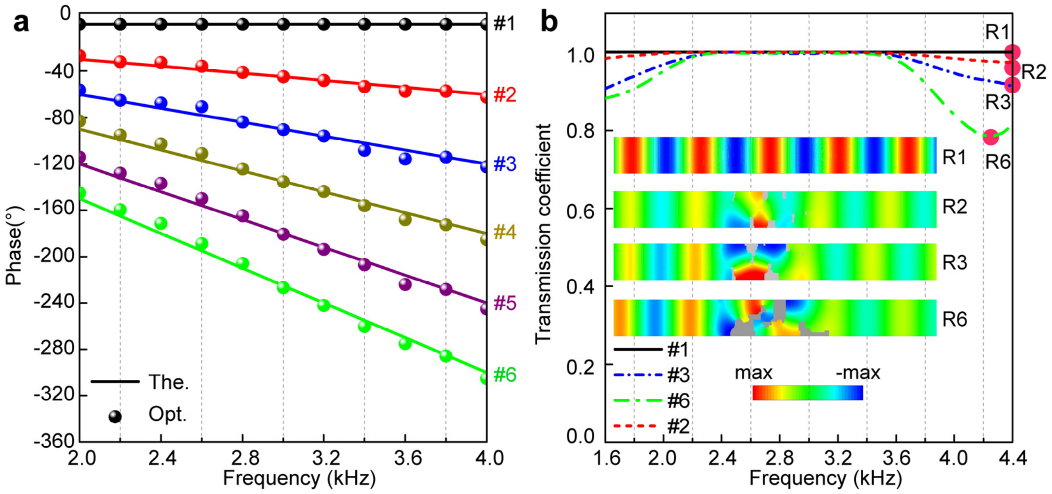

Fig. S8. Phase shifts of all optimized elements for ultra-broadband beam deflection and the representative transmission spectrums. a, Theoretical (The.) and optimized (Opt.) phase shifts. **b**, Transmission spectra of optimized Elements #1, #2, #3 and #6. The insets illustrate the steady state responses under incident plane waves.

In general, unlike the symmetric structure, an asymmetric metasuface element can exhibit strong anisotropy. To confirm and characterize this property, we check the impedance matrices [13] $\begin{bmatrix} Z_{11} & Z_{12} \\ Z_{21} & Z_{22} \end{bmatrix}$ of the optimized Elements #1-#5 in Figs. S9a-e. On account of the high symmetry, Element #1 has the same values of Z_{11} and Z_{22} as expected. However, other Elements #2-#5 have the inconsistent profiles of Z_{11} and Z_{22} profiles, indicating the typical bi-anisotropy [13]. This difference enlarges in the relatively high- or low-frequency ranges. Moreover, as illustrated in Figs. S9f-g, only the phases of the reflection for the forward and backward directions are different, clearly marking the bi-anisotropy [13] of the metasurface element as well. To further show the holistic bi-anisotropy, we calculate the bi-anisotropy extent of all elements in Fig. S9h. The obvious bi-anisotropy can be observed for all optimized Elements #2-#6. Interestingly, the bi-anisotropy feature occurs discontinuously rather than in the entire frequency range. This means that the ultra-broadband beam deflection does not need strong and continuous bi-anisotropy.

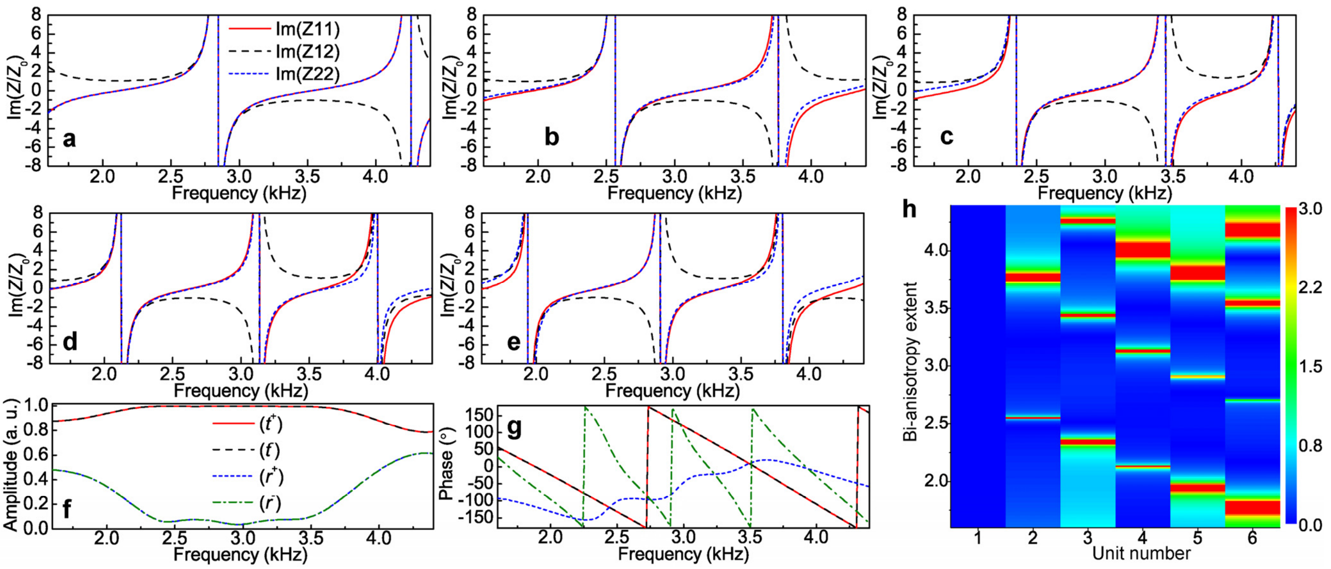

Fig. S9. Bi-anisotropy characterizations of all optimized elements for ultra-broadband beam deflection. a-e, Impedance matrices of optimized Elements #1 (a), #2 (b), #3 (c), #4 (d) and #5 (e) in Fig. 2. **f-g**, Amplitudes and phases of the transmission and reflection coefficients of Element #6 in Fig. 2. **h**, Bi-anisotropy extent $|Im(Z_{11}) - Im(Z_{22})|$ of every optimized element in Fig. 2.

To confirm the multiple-scattering effect in the optimized elements, we compare the traditional retrieved transfer matrix using the four-microphone method [1] with the one under the single-scattering assumption, see Fig. S10. Similar with the bi-anisotropy shown in Fig. S9, the difference in Figs. S10c-f becomes more obvious in the relatively high-frequency or low-frequency range. The difference between the two transfer matrices suggests that most optimized elements cannot be characterized by the single-scattering theory, indirectly supporting the existence of multiple scattering.

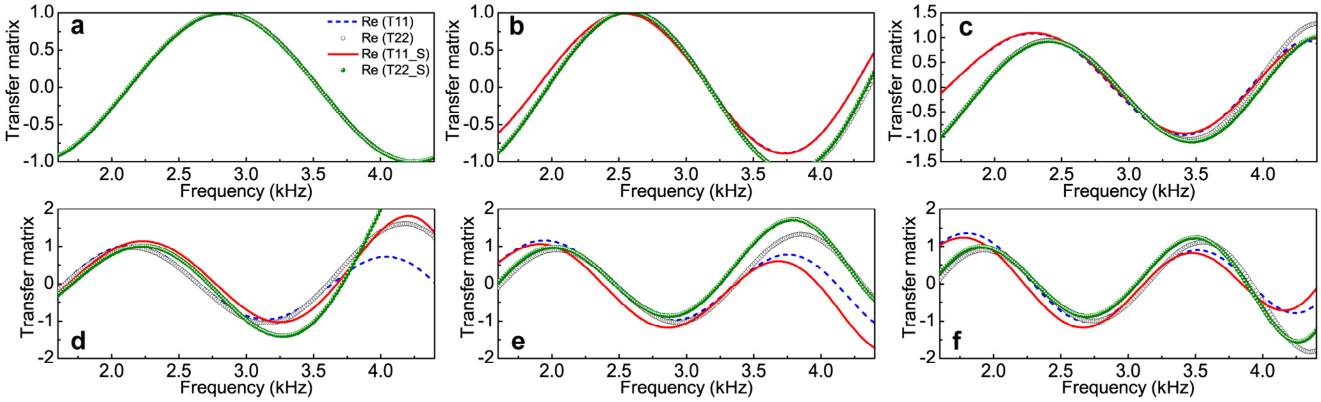

Fig. S10. Characterization of the transfer matrix for ultra-broadband beam deflection. Profiles of transfer matrix are calculated by the traditional retrieved method (T11, T22) or under the single-scattering assumption (T11_S, T22_S) for optimized Elements #1 (a), #2 (b), #3 (c), #4 (d), #5 (e) and #6 (f) in Fig. 2, respectively.

To further show the multiple-scattering effect, we also study the effective index of elements deduced by the single-scattering assumption or traditional retrieved method. The results in Fig. S11 highlight the difference between two kinds of indices, namely, the multiple scattering can effectively modulate the dispersion of the optimized elements in Fig. S7. In some narrow frequency ranges, the difference is so weak that the effect can be neglected. In other words, the single-scattering effect can ensure that the element satisfies the special dispersion requirement only within a narrow bandwidth. This further explains the narrow-band characteristics of the existing space-coiling [12] and Helmholtz-resonator [12] metasurfaces because of their single-scattering nature.

To quantitatively characterize the multiple scattering, we present the multiple-scattering factors of the optimized elements in Figs. S11g-i. It is observed that optimized Elements #4 and #6 have stronger multiple scattering than the others do. Both Elements #2 and #3 exhibit very weak multiple scattering. Accordingly, the multiple scattering affects the effective index of every optimized element at varying levels. We believe that only the inverse design can systematically cope with these diverse forms of multiple scattering for a metasurface on demand.

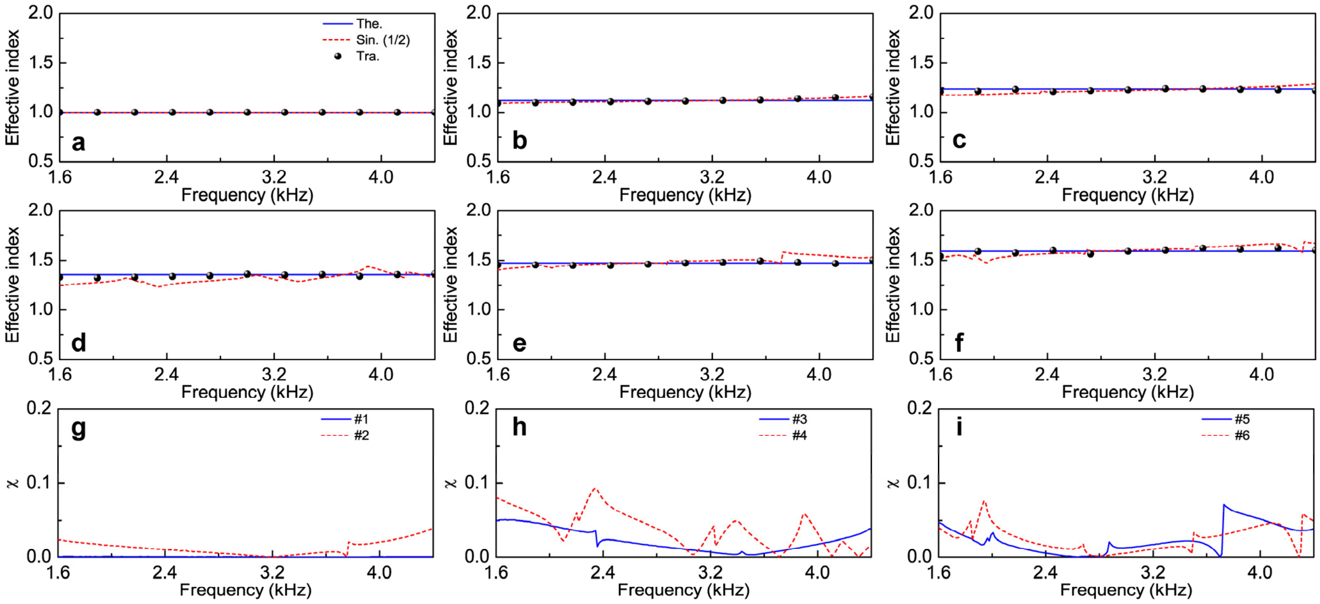

Fig. S11. Evidence of multiple scattering in the inversely designed metasurface for ultra-broadband beam deflection. a-f, Results show the comparison of the theoretical (The.), traditional retrieved (Tra.) and single-scattering (Sin.) induced effective indices of optimized Elements #1 (a), #2 (b), #3 (c), #4 (d), #5 (e) and #6 (f) in Fig. 2, respectively. The index from the single-scattering approximation is calculated based on the division of the element at location 1/2 shown in Fig. S4. **g-i**, Multiple-scattering factors of optimized Elements #1-#2 (g), #3-#4 (h), #5-#6 (i) in Fig. 2.

S10. Supplementary results for ultra-broadband beam deflection

The assumption of a hard wall boundary is commonly used in the field of acoustic metamaterials to transmit sound through air. To demonstrate the accuracy of this assumption, we present in Figs. S12(a-b) simulated wave responses of each metasurface element to steer the beam under the incident plane wave. It is clearly that the wavefronts in both cases are almost identical, with the exception of a very weak difference in the local pressure values at the air-solid interface. Figures S12(c-d) depict the pressure fields of the entire metasurface under the incident plane wave. Similarly, the angle of refraction and transmission efficiency are very close to each other in the two cases. Therefore, in this case, the interaction between air and solid parts can be neglected.

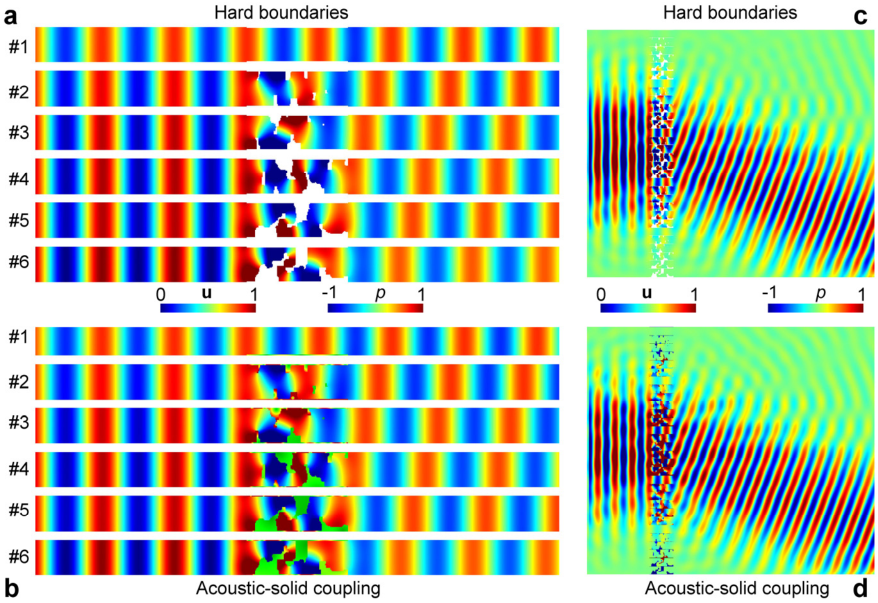

Fig. S12. Simulations of a model with boundaries or acoustic-solid coupling for beam deflection. a-b, Pressure and displacement fields of six metasurface elements under incident plane waves at 4000 Hz. c-d, Pressure and displacement fields of anomalous refraction at 4000 Hz under incident plane waves.

To verify the inversely designed metasurface for ultra-broadband beam deflection, we present theoretical and numerical full-wave simulations in Fig. S13. As displayed in Figs. S13a and S13b, for six operating frequencies, the simulated negative refraction without considering any loss can completely match with the theoretical one. When the relative narrow input waves are incident on the metasurface, the refraction angle is highly consistent with the theoretical value of 20.2°, see Figs. S13b. If the incident waves become wider, however, evident near-field scattering occurs at 2000 Hz and 2600 Hz, see Fig. S13c. In the topology optimization, the phase shifts at 4000 Hz and 2000 Hz should be 60° and 30°, respectively. So, the continuous phase exists at the interface between two periodic combined structures at 4000 Hz. As a result of the phase shift of 180° at 2000 Hz, the phase at the same interface is distinctly discontinuous, which results in the above near-field scattering. Note that this drawback is determined by the generalized Snell's law rather than the present design methodology. Certainly, in the future work we can employ more elements with smaller element-size to perform topology optimization. For different operating frequencies, we can accordingly adjust the location and the number of the optimized elements to get a continuous phase distribution.

To account for thermo-viscous loss in simulations, a thermoacoustic interface is used to compute changes in acoustic pressure, velocity, and temperature. The interface is necessary for accurate calculation of the acoustic field in geometries of small dimensions, therefore the thermo-acoustics domain is introduced into the area with element-cells containing complex acoustic channels. We then introduce acoustic-thermoacoustic boundary nodes to couple the thermoacoustic domain with the acoustic domain. Viscosity and thermal conductivity near solid walls become important because they create a viscous and thermal boundary layer where losses are significant. Therefore, the boundary layers have a grid with distribution of dense elements in the normal direction along specific boundaries.

Calculation of thermo-viscous acoustic in the frequency-domain simulates the propagation of compressible linear waves in a general-purpose viscous and heat-conducting fluid by solving the fully linearized Navier-Stokes (momentum), continuity, and energy equations. The continuity equation of the thermoacoustic calculation in the frequency domain is

$$i\omega\rho_t + \nabla\cdot(\rho_0\mathbf{u}_\mathrm{t}) = 0\,, \tag{20}$$

where ρ_t is the total density, and $\mathbf{u}_\mathrm{t}$ is the total acoustic velocity.

The fully linearized Navier-Stokes (momentum) equation has the form

$$i\omega\rho_0\mathbf{u}_\mathrm{t} = \nabla\cdot\left[-p_t\mathbf{I} + \mu(\nabla\mathbf{u}_\mathrm{t} + (\nabla\mathbf{u}_\mathrm{t})^\mathrm{T}) - (\frac{2}{3}\mu - \mu_\mathrm{B})(\nabla\cdot\mathbf{u}_\mathrm{t})\mathbf{I}\right], \quad (21)$$

here μ is dynamic viscosity, and μ_B is bulk viscosity. The term on the right is divergence of the stress tensor.

The energy conservation equation is

$$i\omega(\rho_0 C_\mathrm{p} T - T_0\alpha_0 p) = -\nabla\cdot(-k\nabla T) + Q, \quad (22)$$

where C_p is heat capacity at constant pressure, and k is thermal conductivity, and α_0 is coefficient of thermal expansion (isobaric), and Q is a possible heat source.

Some previous studies have revealed that the viscous loss induced by the near-wall viscosity effect usually occurs in the element with very narrow air channels [14]. The thermal-viscous losses can result in a reduction in the transmission efficiency and a shift in the Fabry-Perot resonance frequency. Fortunately, we can observe from Figs. S13d the perfect beam deflection, clearly demonstrating the loss effects are rather limited in our case and our metasurfaces can offer a certain degree of robustness and immunity to the thermal-viscous loss.

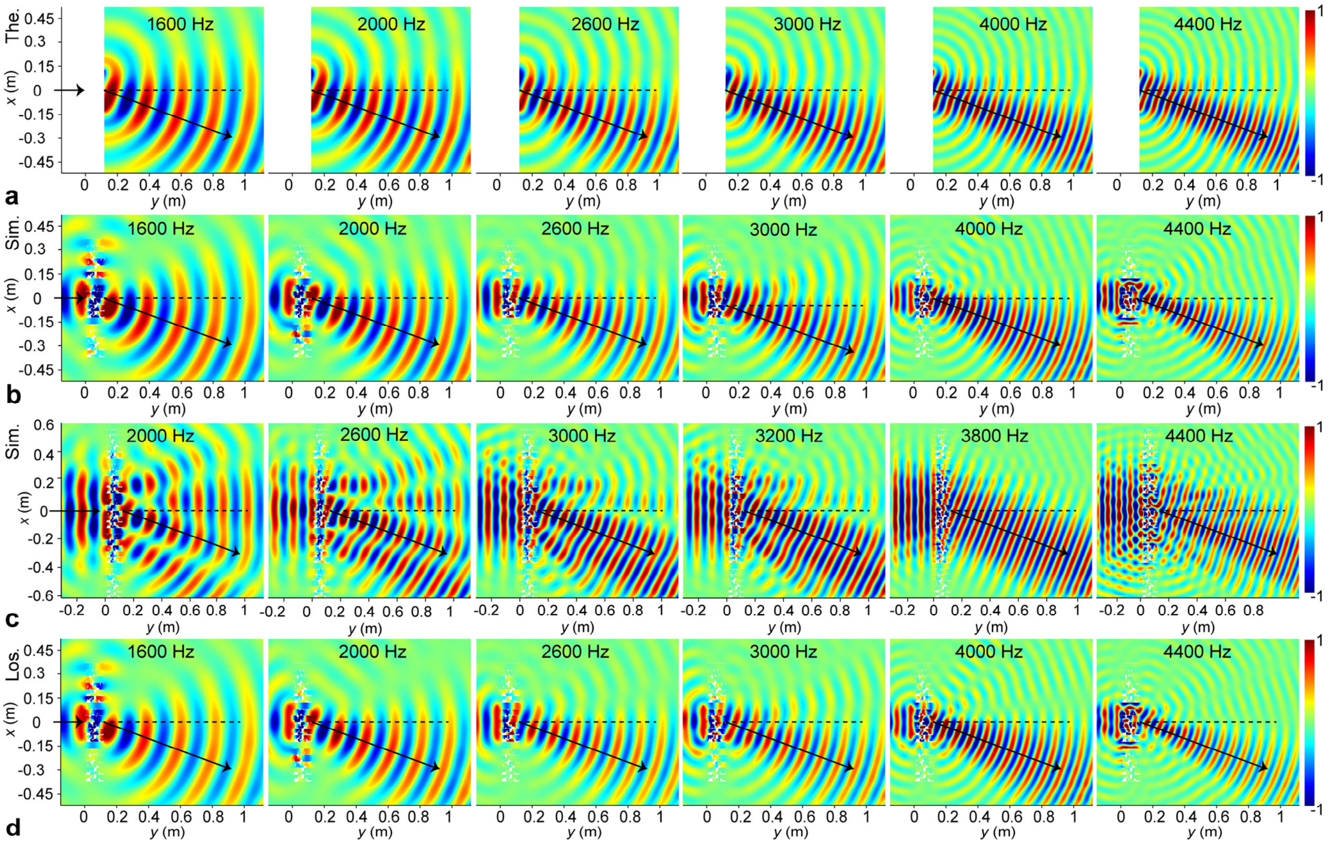

Fig. S13. Theoretical and simulated ultra-broadband beam deflection. a, Theoretical (The.) beam deflection considering the perfect phase distribution and the ideal total-transmission. **b-c**, Simulated (Sim.) acoustic amplitude fields under the incident plane wave of one-period (b) or three-period (c) width without the thermal-viscous loss based on the inversely designed metasurface in Fig. 2. **d**, Simulated acoustic amplitude fields under the incident plane wave beam of one-period width with the thermal-viscous loss (Los.).

S11. Characterizations of optimized elements for ultra-broadband focusing

In view of the same non-dispersive nature, except Element #1, metasurface elements for ultra-broadband focusing can be designed by using the topology optimization in which the required phase shift of every element is relative to its previous element. Like the optimization for beam deflection, the inverse design can effectively reduce the search difficulty and computing time. As shown in Fig. S14, optimized Elements #4-#7 have the same topological feature, i.e., four asymmetric solid blocks with curved air channels. As the required index increases, the minimal air channel becomes narrower. Overall, the optimized elements have both the cavity-based and space-coiling topological features. These novel asymmetric structures demonstrate, once again, that only the inverse design can overcome the limitations of the empirical design and bring about the prominent wavefront property.

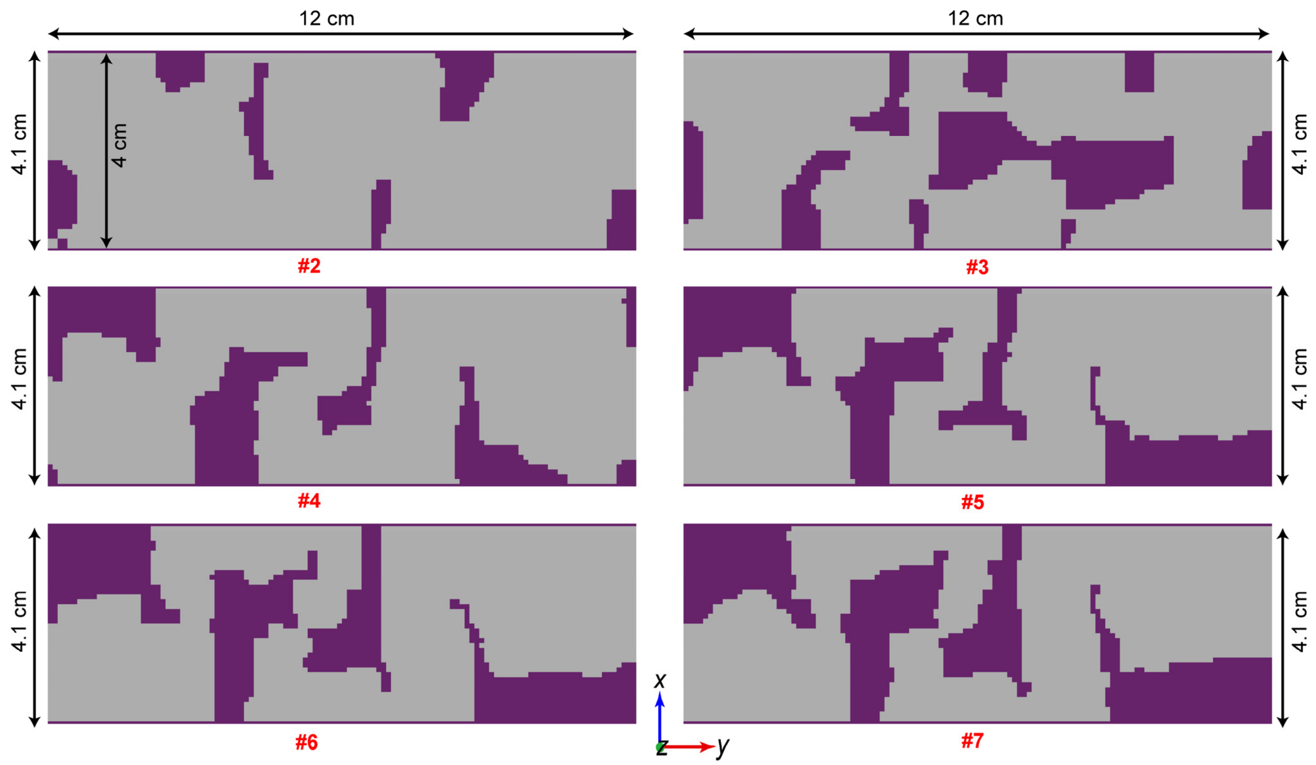

Fig. S14. Topologies of all optimized elements for ultra-broadband focusing. The purple and grey parts represent the solid and air elements, respectively.

As depicted in Fig. S15a, the phases of seven optimized elements nicely match with the theoretically required ones. Like the results in Fig. S8a, the perfectly matched phase shifts can be generated through reducing the demand on transmission. Alternatively, narrowing the target frequency range in the topology optimization can also further improve the matching degree.

To check the possible existence of FP resonance effect, we calculated the transmission spectra of Elements #2, #3 and #7 and found that no FP resonance occurs for Elements #2 and #7. However, the exact frequency at the minimal transmission (38.2%) of Element #3 is 1880 Hz which is very close to its theoretical value f_{FP}=1904 Hz, keeping the very small relative difference of 1%. In this case, Element #3 indeed induces the FP resonance.

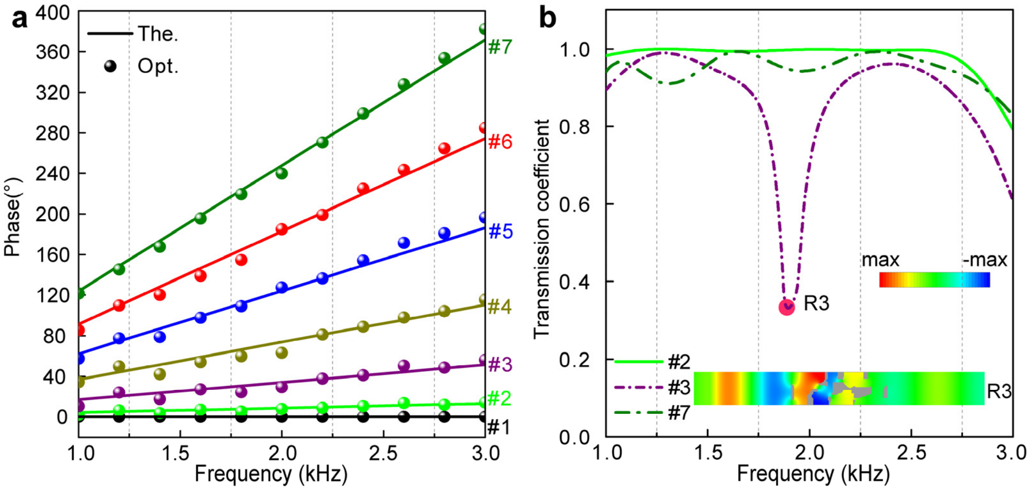

Fig. S15. Phase shifts of all optimized elements for ultra-broadband focusing and the representative transmission spectrums. a, Theoretical (The.) and optimized (Opt.) phase shifts. **b**, Transmission spectra of the optimized Elements #2, #3 and #7. The inset illustrates the steady state response under the incident plane waves.

Now that the optimized elements in Fig. S14 exhibit highly asymmetric characteristics, it is conceivable that they would carry the bi-anisotropy property. Figure S16 clearly shows the difference between the profiles of Z_{11} and Z_{22}, demonstrating the typical bi-anisotropy of the optimized elements in Fig. S16. We can find that the optimized Element #3 has the strongest bi-anisotropy compared with others. In contrast, both the optimized Elements #2 and #4 have very weak bi-anisotropy, while the bi-anisotropy extents of the optimized elements are relative larger. These various bi-anisotropy distinctions make the design of the metasurface for simultaneously achieving ultra-broadband focusing and high overall transmission extremely challenging.

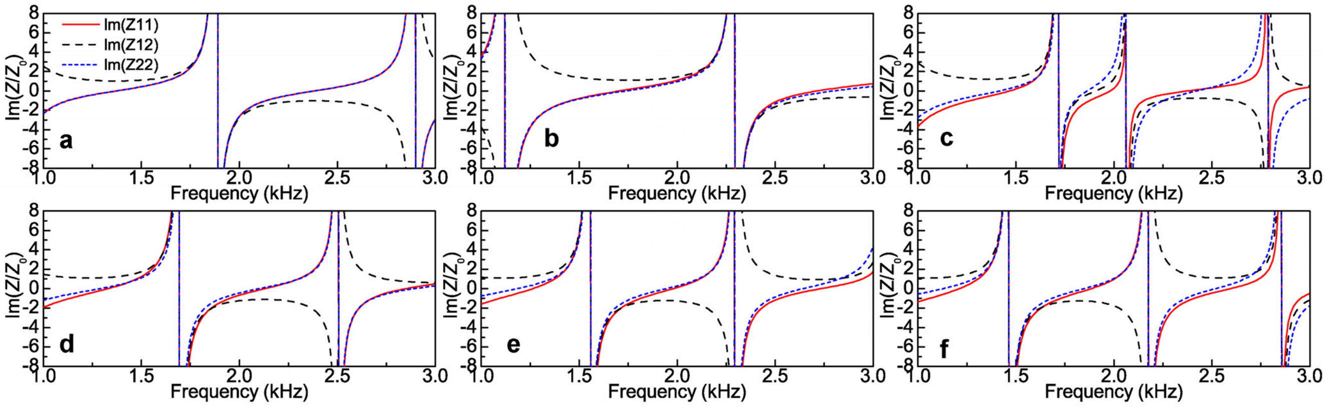

Fig. S16. Impedance matrices of the optimized elements for ultra-broadband focusing. Subgraphs show the results of Elements #1 (a), #2 (b), #3 (c), #4 (d), #5 (e) and #6 (f) in Fig. 3, respectively.

Similarly, we adopt the same approach to verify the multiple scattering effects in the optimized elements for ultra-broadband focusing. We elucidate in Fig. S17 the distinct difference between the transfer matrices through the traditional retrieved method and the single-scattering method. This phenomenon confirms the existence of the multiple scattering in optimized elements in Fig. S14. Apart from Element #2, all the other elements exhibit very strong differences. These six profiles of the transfer matrices can be classified into three categories including optimized Elements #2 (first-class), #3 (second-class) and #4-#7 (third-class), implying that there exit three kinds of topological features which can be also proven by the geometries and topologies shown in Fig. S14. For optimized Elements #4-#7, the difference increases as the frequency increases.

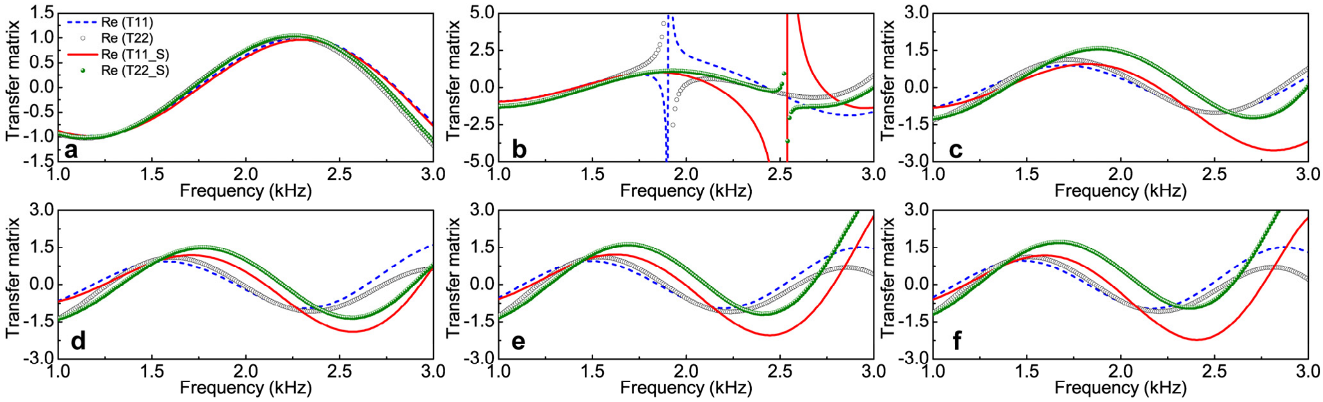

Fig. S17. Characterization of the transfer matrix for ultra-broadband focusing. Profiles of transfer matrix are calculated by the traditional retrieved method (T11, T22) or under (T11_S, T22_S) the single-scattering assumption for optimized Elements #2 (a), #3 (b), #4 (c), #5 (d), #6 (e) and #7 (f) in Fig. 3, respectively.

As for the effective index in these two cases, Figs. S18a-f clearly show the difference between the results based on the single-scattering and traditional retrieved method. Like the tendencies in Fig. S18, six optimized elements can be classified under three categories as well. Multiple scattering in optimized Element #2 is the weakest, supporting a broadband accuracy of index. However, the other five elements possess the apparent inhomogeneous multiple scattering. Therefore, only a few metasurface elements without multiple scattering cannot realize the broadband wave manipulation having the unchanged dispersion. The multiple-scattering factors in Figs. S18g-i quantitatively characterize the multiple scattering of every element. The heterogeneity, irregularity and nonlinearity make the elaborate control of multiple scattering impossible for the artificial or empirical design. Compared with the results shown in Fig. S17, the multiple scattering in Fig. S18 is generally stronger, which means that the non-linear non-dispersive property requires stronger multiple scattering to tailor dispersion than the linear non-dispersive one does.

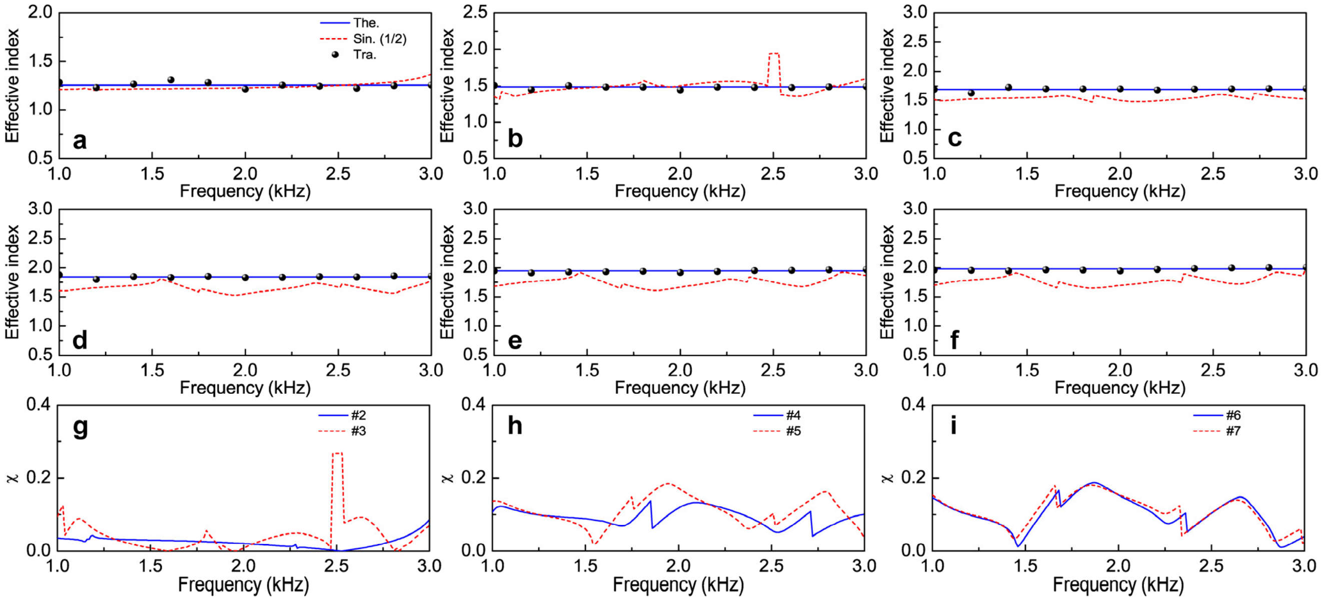

Fig. S18. Evidence of multiple scattering in the inversely designed metasurface for ultra-broadband focusing. a-f, Results show the comparison of the theoretical (The.), traditional retrieved (Tra.) and single-scattering (Sin.) induced effective indices of optimized Elements #2 (a), #3 (b), #4 (c), #5 (d), #6 (e) and #7 (f) in Fig. 3, respectively. The index from the single-scattering approximation is calculated based on the division at location 1/2 shown in Fig. S3. **g-i**, Multiple-scattering factors of optimized Elements #2-#3 (g), #4-#5 (h), #6-#7 (i) in Fig. 3.

S12. Supplementary results for ultra-broadband focusing

To experimentally check the viscous-thermal effect, we experimentally measured the transmission coefficient of two typical metasurface elements intended for focusing. As shown in Fig. S19 below, although the elements contain relatively narrow air regions/channels, the measured transmission coefficients are close to the simulation results without considering any losses or viscous-thermal effect. Both elements can support relatively high transmission in excess of 70% in the range of [1000 Hz, 3000 Hz]. This demonstrates that the viscous-thermal effect is really weak, which does not significantly affect the results shown in Fig. S20. Therefore, only a very long narrow air channel can result in a notable decrease in the transmission, not a narrow local air area.

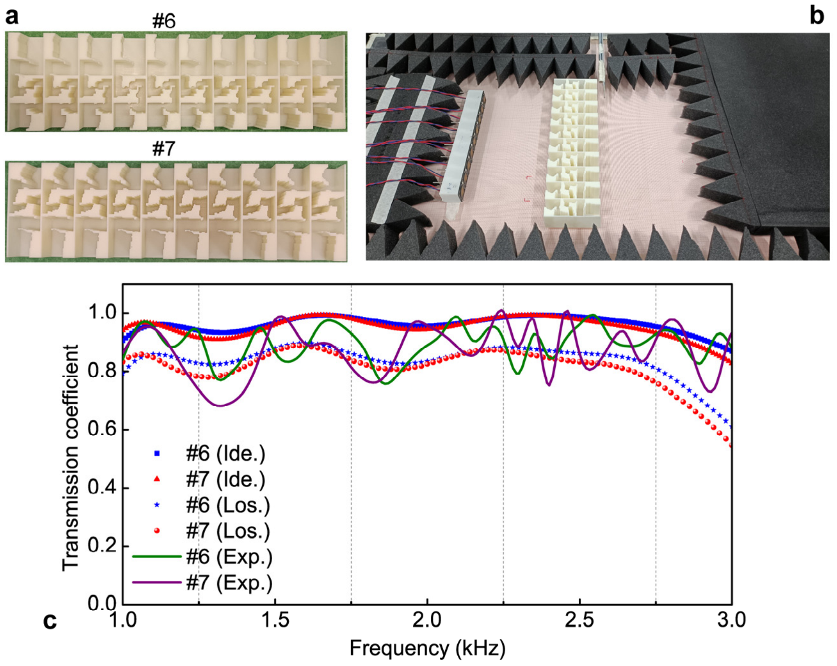

Fig. S19. Simulated and experimental transmission of representative elements #6 and #7 for focusing. a, 3D-printed element samples. b, Experimental setup for testing the transmission of an element. c, Comparison of transmission between ideal lossless simulations (Ide.), viscous-thermal simulations (Los.) and experimental measurements (Exp.).

To show the efficacy of the inversely designed metasurface in Fig. S14, we perform numerical simulations to demonstrate the ultra-broadband focusing in Fig. S20. In theory, the metasurface with the ideal phase distribution and total transmission can give rise to perfect ultra-broadband achromatic focusing as displayed in Fig. S20a. The focus at every representative frequency occurs at the same place regardless of the frequency changes. In comparison, when introducing viscous loss, the inversely designed metasurface can also lead to ultra-broadband achromatic focusing. In principle, the complex geometries and narrow air channels usually induce a certain degree of frequency shift and transmission reduction [14]. Fortunately, as shown in Fig. S20c, the acoustic simulations considering the typical thermal-viscous loss reveal that the present metalens can still maintain its ultra-broadband nature, although the focusing efficiency becomes somewhat lower. We further present in Fig. S20d the normalized amplitude profiles to demonstrate the focusing energy around the desired location F_0=0.2m. Then the acoustic experiment in Fig. S20e is conducted to verify the designed ultra-broadband focusing of the body text.

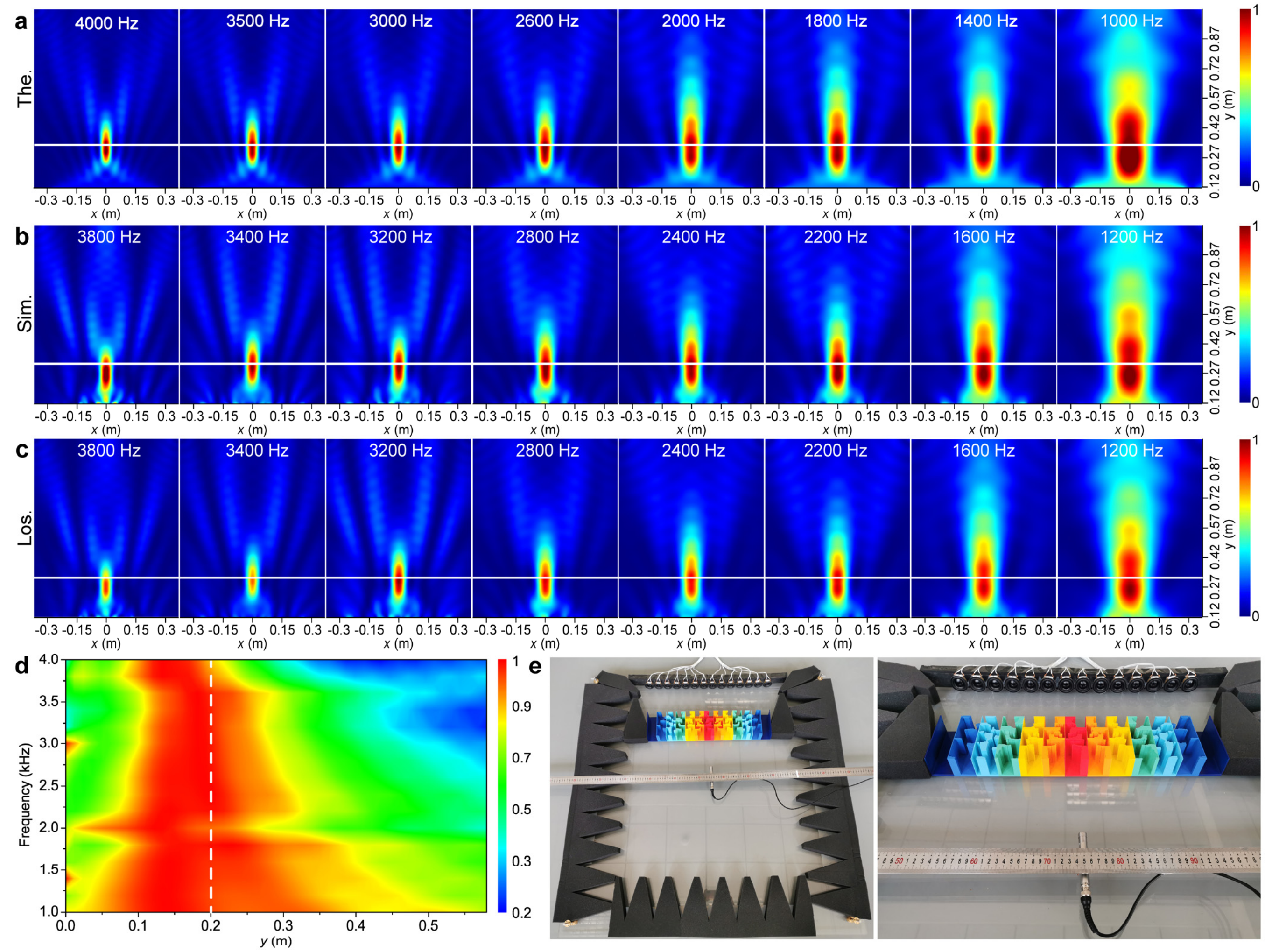

Fig. S20. Theoretical and simulated ultra-broadband focusing. a, Theoretical (The.) focusing considering the perfect phase distribution and the ideal total-transmission. **b**, Simulated (Sim.) acoustic amplitude fields based on the inversely designed metasurface in Fig. 3 without the thermal-viscous loss. **c**, Simulated acoustic amplitude fields with the thermal-viscous loss (Los.). For the same frequency, the simulated fields without and with the thermal-viscous loss use the same normalization scale with the same maximal pressure amplitude. Results at different frequencies adopt the different normalization scales. **d**, Simulated normalized acoustic pressure amplitude profiles without the thermal-viscous loss at x=0 along the y-direction within [1000 Hz, 4000 Hz]. The x-axis value means the distance away from the metasurface. **e**, Experimental setup. The loudspeaker acting as the acoustic plane wave source is placed 0.15 m away from the metasurface surrounded by the acoustic absorbing foams to avoid reflections in experiments, while the mounted microphone measured the acoustic field by moving in the scanning area.

S13. Characterizations of optimized elements for ultra-broadband levitation

To generate single-side acoustic levitation, we perform topology optimization of a series of elements to construct a 3D metasurface for realizing the required rigorous phase distribution, see Fig. S21a. To get the bottle beam for levitation, the metasurface should simultaneously support the superposition of the phase distribution for focusing and an additional phase screen where a central circular region (pink elements) is out of phase with the outer regions (green elements). Such two kinds of elements, theoretically, have different dispersion features, see Fig. 4b in the main text. With the square-symmetry assumption, the 3D inversely designed metasurface can be constructed by 28 different elements and their symmetrical ones. In view of the complex dispersion pattern, the metasurface elements for ultra-broadband levitation are designed by adopting

the topology optimization in which the required phase shift of every element is solely relative to Element #27 that is defined as the reference structure.

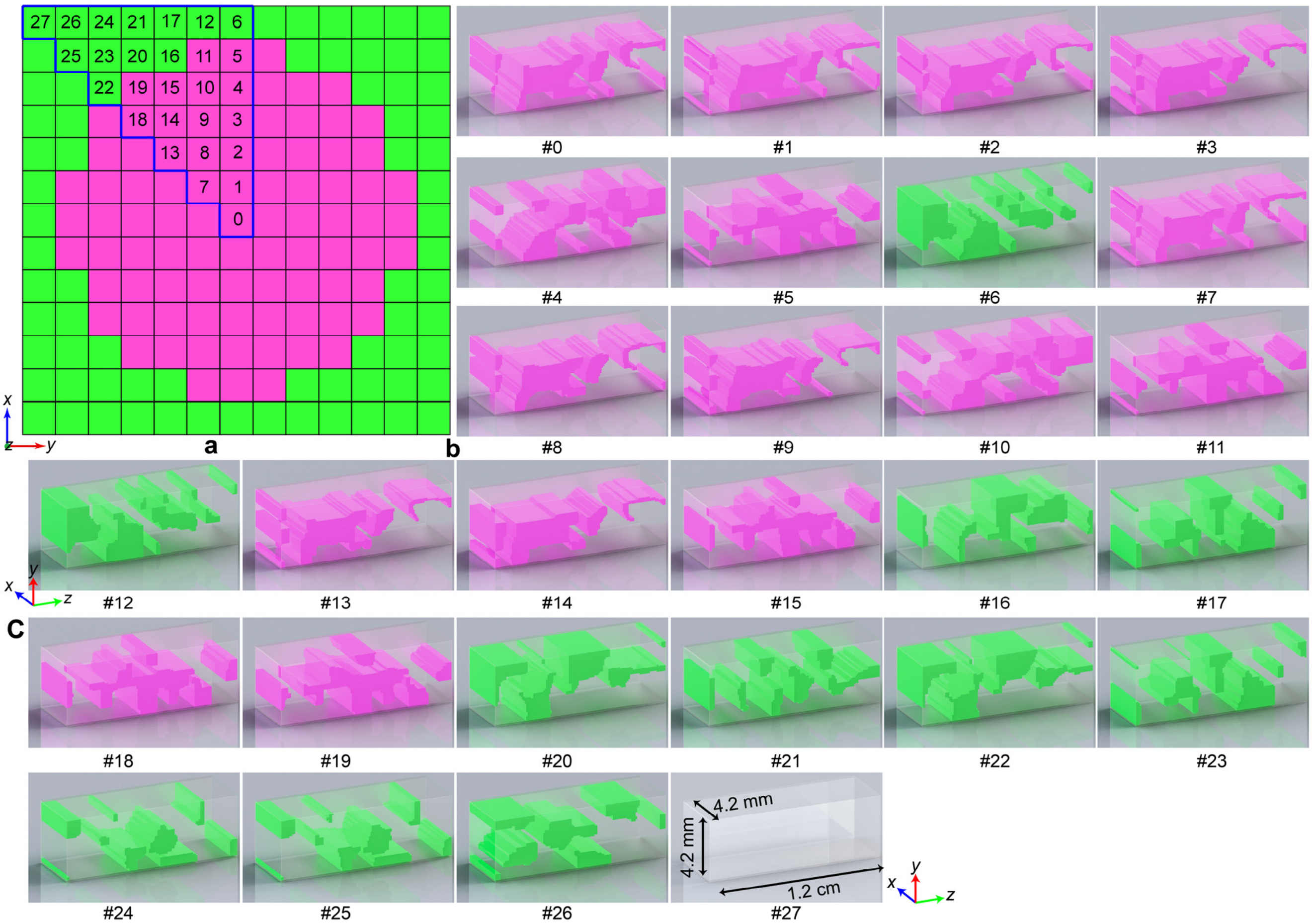

Fig. S21. 3D sketches of all optimized elements for ultra-broadband levitation. a, 2D Binary distribution of all 13×13 elements. b-c, Render images of all elements. The upper and lower solid edges with the width of 100 μm are marked by the transparent color to clearly show the inner geometries of the metasurface elements. The colored components express the solid materials. Optimized Elements #0-#5, #7-#11, #13-#15, #18 and #19 are dispersive. Optimized Elements #6, #12, #16, #17 and #20-#27 are non-dispersive.

In fact, the 3D optimized elements in Figs. S22b and S22c are assembled by using the 2D optimized elements shown in Figs. S23a and S23b. Figs. S22 and S23 clearly show the corresponding topologies and geometries. All these brand-new 3D and 2D elements are asymmetric and complex, which can hardly be achieved by artificial design. Their topological feature is totally different from the existing Helmholtz-resonator and space-coiling based metasurface elements, i.e., 1) multiple discretized asymmetric solid cylinders; 2) several local cavities; 3) highly asymmetric distributed cylinders; and 4) curved air channels. In macro sense, the dispersive elements can be divided into three subclasses: structures with a simply-connected air region and one cavity (Elements #0-#3, #7-#9, #13 and #14), structures with bi-connected air region (elements #4 and #10) and structures with a simply-connected air region and three cavities (Elements #5, #11, #15, #18 and #19). Their similar configurations imply that the optimized topological features can satisfy a range of dispersive index requirement even for the complex wave manipulation. Similarly, the non-dispersive elements can also be divided into five subclasses: structures with a small cavity (Elements #24 and #25), structures with no visualized cavity (Elements #17, #23 and #26), structures with a small cavity and a curved air channel at the left side (Elements #6, #12, #20 and #22) and analogous labyrinth structure (Element #21).

Altogether, the novel topological features of the optimized elements presented in Figs. S7, S14, S21 and S22 offer a new way for constructing the metasurfacecs for the complex ultra-broadband wave manipulation which is unachievable by the existing metasurfaces. We believe that the inverse-design methodology would be the necessary way for the customized metasurface devices in the future.

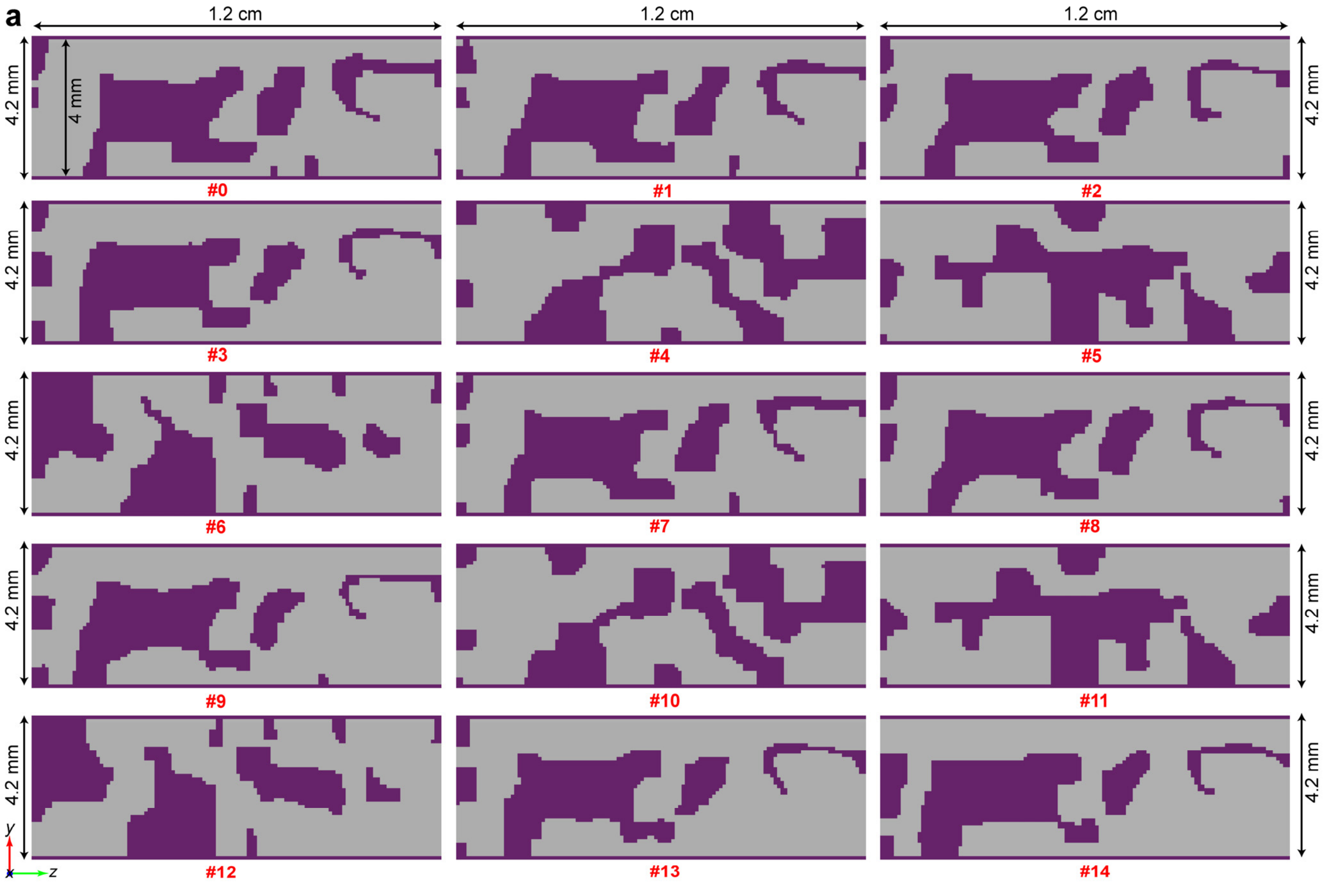

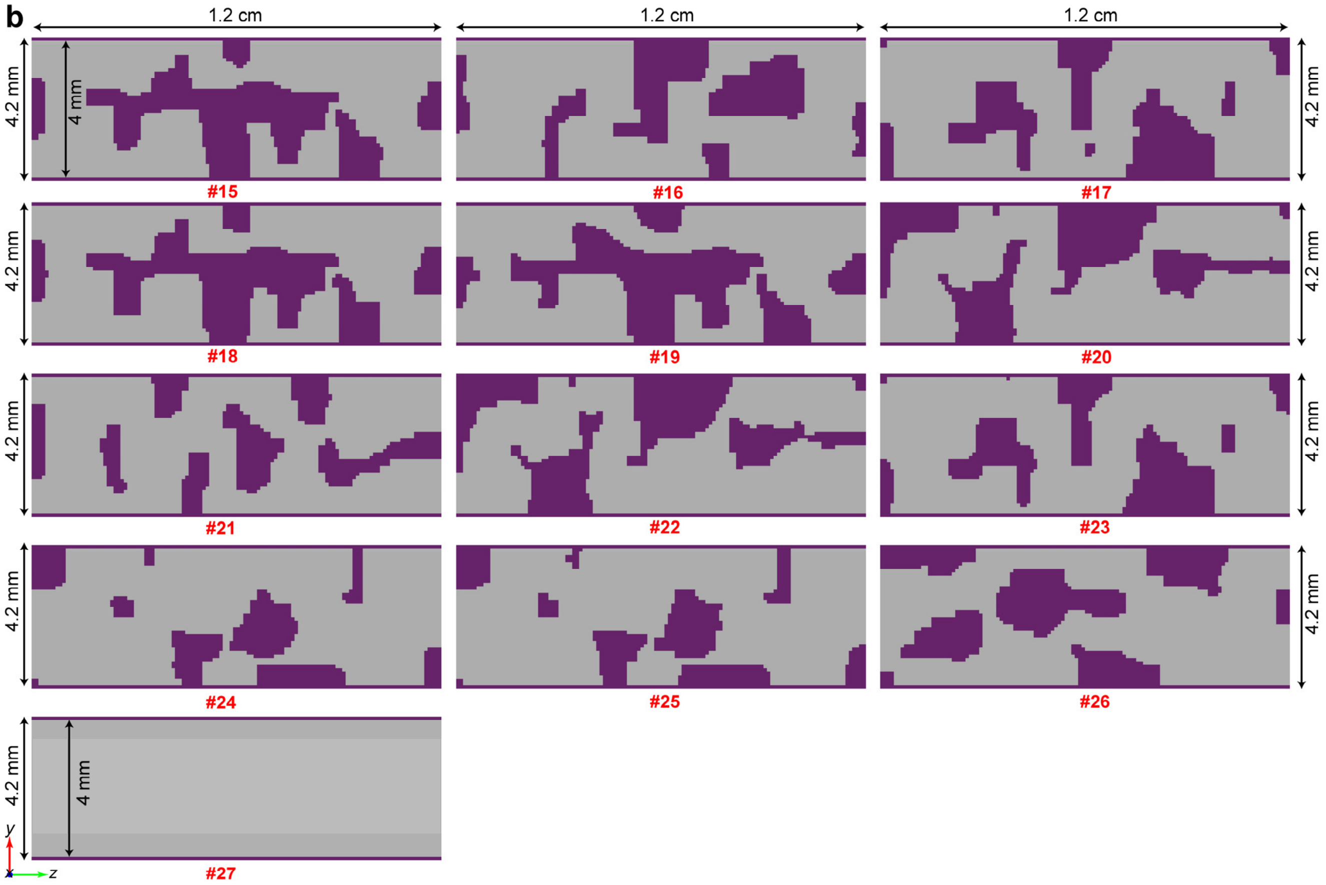

Fig. S22. Topologies of all optimized elements for ultra-broadband levitation. Subgraphs show the 28 representative Elements #0-#27 in Fig. S21. The purple and grey parts represent the solid and air elements, respectively.

As illustrated in Fig. S23, the phases of all optimized elements are highly consistent with the theoretically required ones. We can clearly recognize the dispersive and non-dispersive elements. Of course, the perfect phase matching inevitably results in a degradation of the transmission. Nevertheless, we will show that the current combination of the phase matching and the transmission of metasurface will not substantially deteriorate the ultimate levitation.

To check the existence of FP resonance effect, we show the calculated transmission spectra of Elements #1, #4 and #17 in Fig. S20c. It follows that the FP resonance occurs for elements #4 and #17. Element #17 has the FP resonance effect at 31.4 kHz which is about 6% smaller than theoretical value f_{FP}=33.39 kHz, when the transmission decreases to 18%. Element #4 also has the FP resonance effect at 18.15 kHz with the theoretical value f_{FP}=18.11 kHz, while reaching the transmission as low as 4%. But Element #1 has no FP resonance effect because of the relatively high transmission.

Results in Figs. 8b, 15b and 23b suggest that typical FP resonance only occur in some elements when the transmission is low at certain frequencies. This however would not happen when the element has a high transmission. Fortunately, even when FP resonance effect takes place, it usually appears within narrow frequency ranges. Therefore, the three finally achieved wave functionalities are basically not affected by this effect in our case, as demonstrated by the nearly ideal performance shown in Figs. 2D, 3D, 4E and 4F.

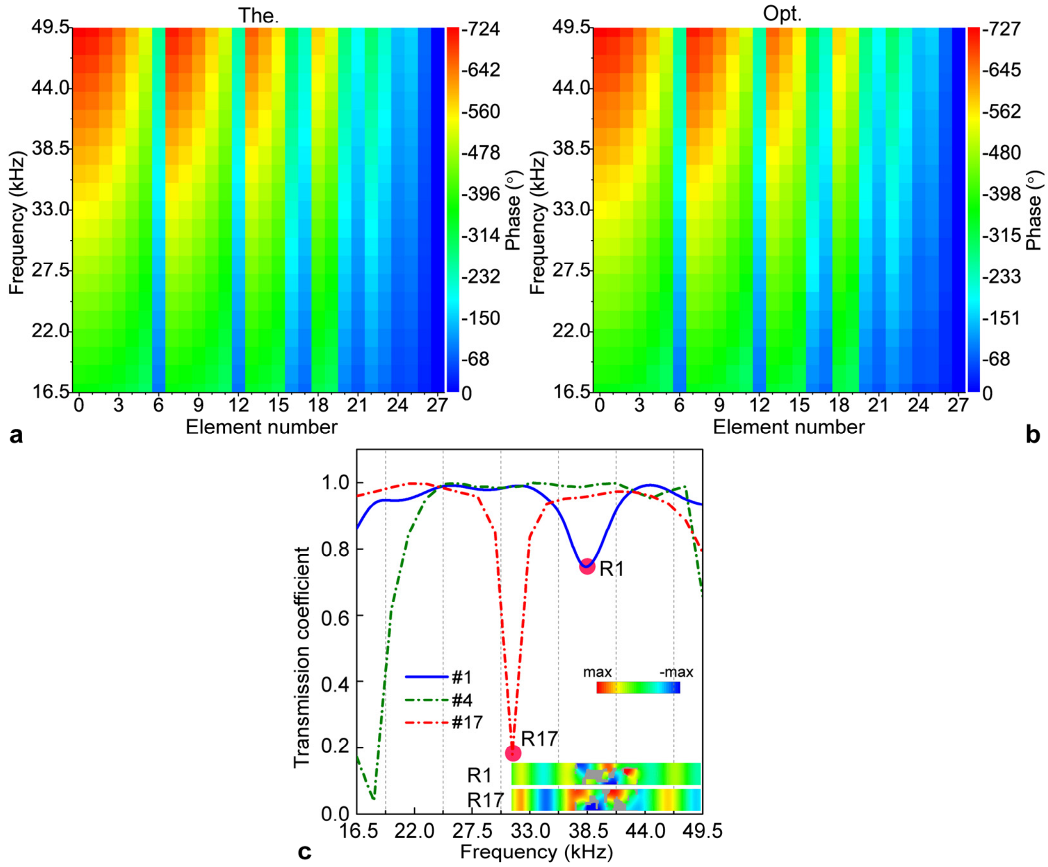

Fig. S23. Phase shifts of all optimized elements for ultra-broadband levitation and the representative transmission spectrums. a-b, Theoretical (The.) and optimized (Opt.) phase shifts. **c**, Transmission spectra of the optimized Elements #1, #4 and #17. The insets illustrate the steady state responses under the incident plane waves.

To further show the effect of the asymmetric topology on wave motion, we check in Fig. S23 the impedance matrices for the optimized asymmetric elements shown in Figs. S21 and S22. Compared with the results for ultra-broadband beam deflection and focusing, ultra-broadband levitation involves stronger bi-anisotropy. Interestingly, we find from Fig. S24 that the non-dispersive elements have stronger bi-anisotropy than that of the dispersive ones. As a result, a perfect ultra-broadband metasurface should be elaborately designed for the diverse bi-anisotropy for every encompassing element and non-linear distribution of bi-anisotropy for the element.

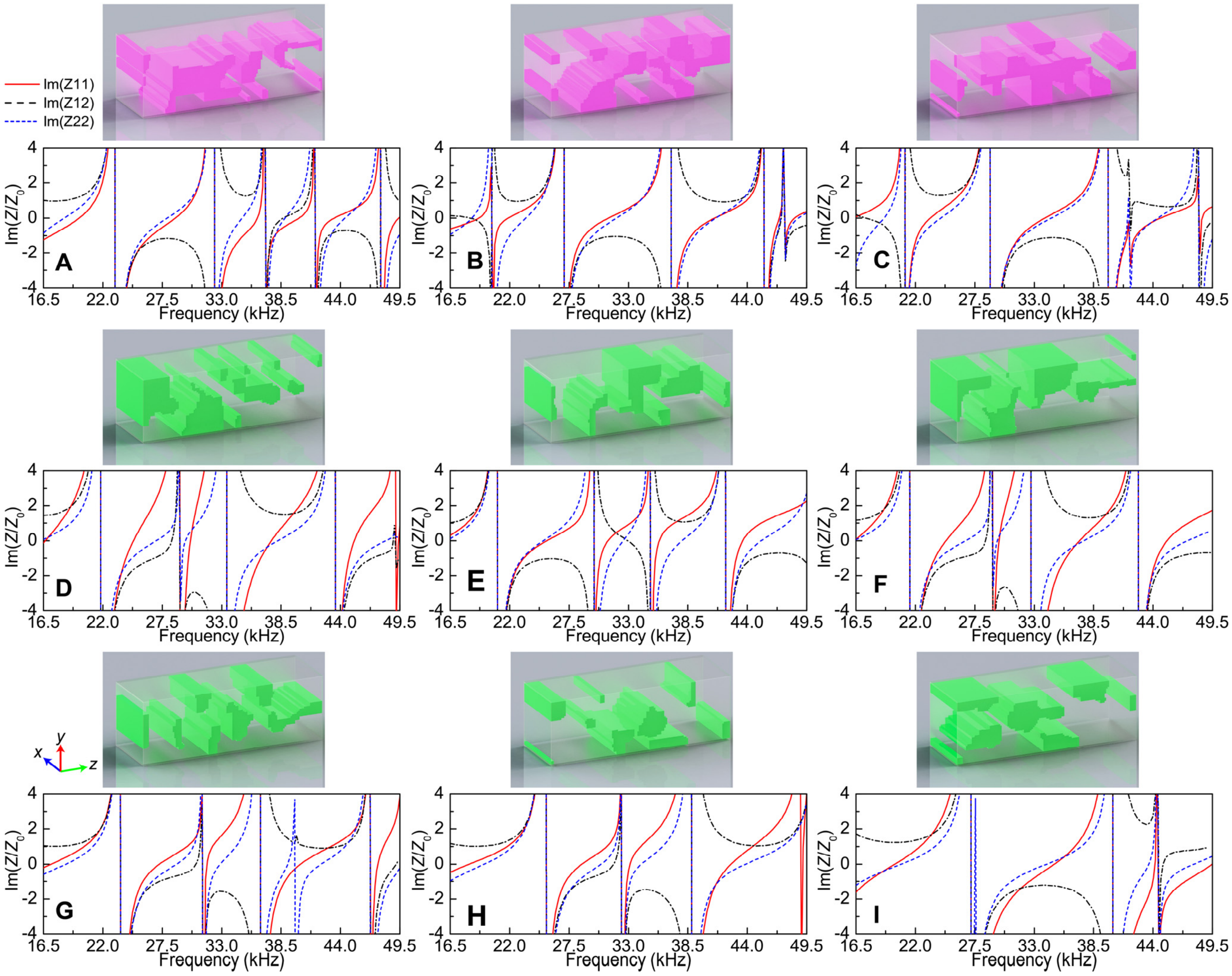

Fig. S24. Impedance matrices of the representative optimized elements for ultra-broadband levitation. Subgraphs show the results of Elements #3 (**a**), #4 (**b**), #5 (**c**), #6 (**d**), #16 (**e**), #20 (**f**), #21 (**g**), #24 (**h**) and #26 (**i**) in Fig. S21, respectively. Representative optimized Elements #3, #4 and #5 are dispersive. Representative optimized Elements #6, #16, #20, #21, #24 and #26 are non-dispersive.

We have also noticed obvious multiple scattering in the optimized elements for ultra-broadband beam deflection and focusing. Indeed, the results shown in Fig. S25 also demonstrate the existence of the multiple scattering for ultra-broadband levitation. Comparing with the other elements, the optimized Element #4 has the strongest multiple-scattering effect and the largest dispersion. Generally, more peaks will induce stronger multiple scattering.

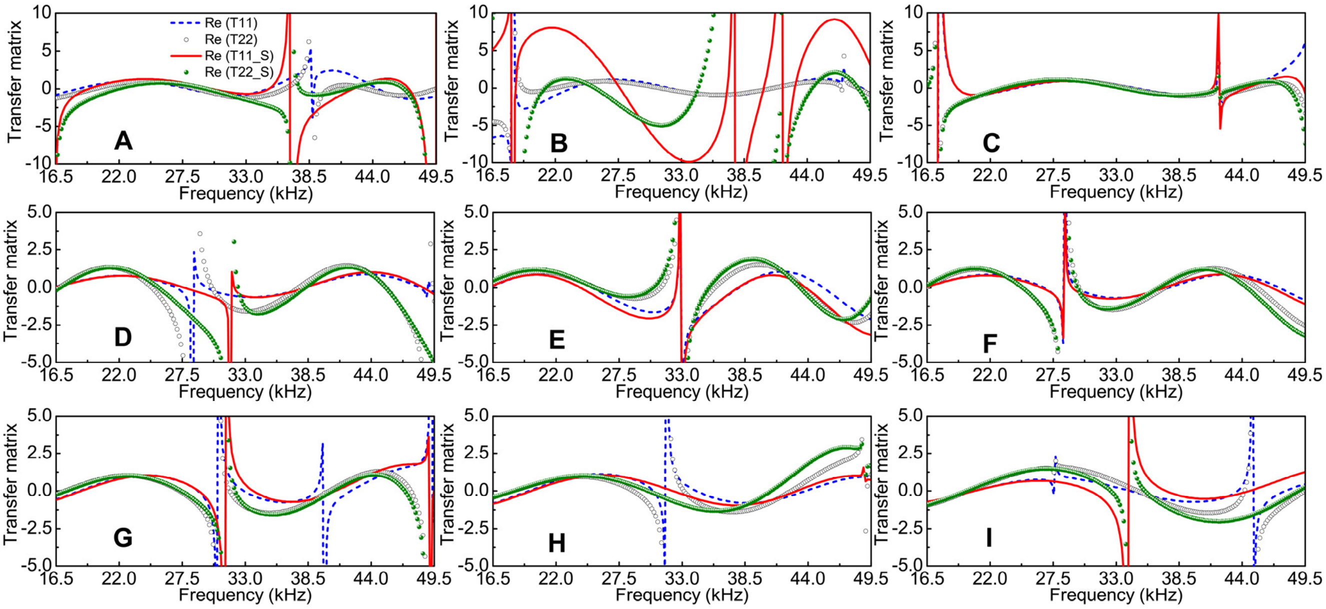

Fig. S25. Characterization of the transfer matrix for ultra-broadband levitation. Profiles of the transfer matrix are calculated by the traditional retrieved method (T11, T22) or under (T11_S, T22_S) the single-scattering assumption for optimized Elements #3 (**a**), #4 (**b**), #5 (**c**), #6 (**d**), #16 (**e**), #20 (**f**), #21 (**g**), #24 (**h**) and #26 (**i**) in Fig. S21, respectively. Representative optimized Elements #3, #4 and #5 are dispersive. Representative optimized Elements #6, #16, #20, #21, #24 and #26 are non-dispersive.

To further assess the multiple scattering for ultra-broadband levitation, we present in Fig. S26 the effective indices induced by the traditional retrieved method and the single-scattering assumption. As illustrated in Fig. S26, the inversely designed metasurface for ultra-broadband levitation consists of two types of elements, i.e., one is non-linear non-dispersive and the other one is non-linear dispersive. All these optimized elements support multiple scattering to a certain extent, see Figs. S26a-i. On the whole, the required theoretical index is not positively correlated with the multiple scattering extent. For example, optimized Element #26 with the small index has stronger multiple scattering than Elements #6, #21 and #24. In fact, the division location of computational model in Fig. S4 can affect the obtained index of element. This effect will be intensified if there is a cavity or solid block at the division location. Combining the results in Fig. 5i of the body text and Figs. S26a-b, we could deduce that the dispersive elements may possess stronger multiple scattering than the non-dispersive ones. Especially for Element #0, multiple scattering is the most significant. In some local frequency ranges, the single scattering can lead to relatively accurate effective index. In other words, an optimized element may show obvious single-scattering and multiple-scattering behavior in different frequency ranges. It is the complex multiple scattering that makes the dispersion control of metasurface for ultra-broadband levitation much more difficult than for the aforementioned beam deflection and focusing.

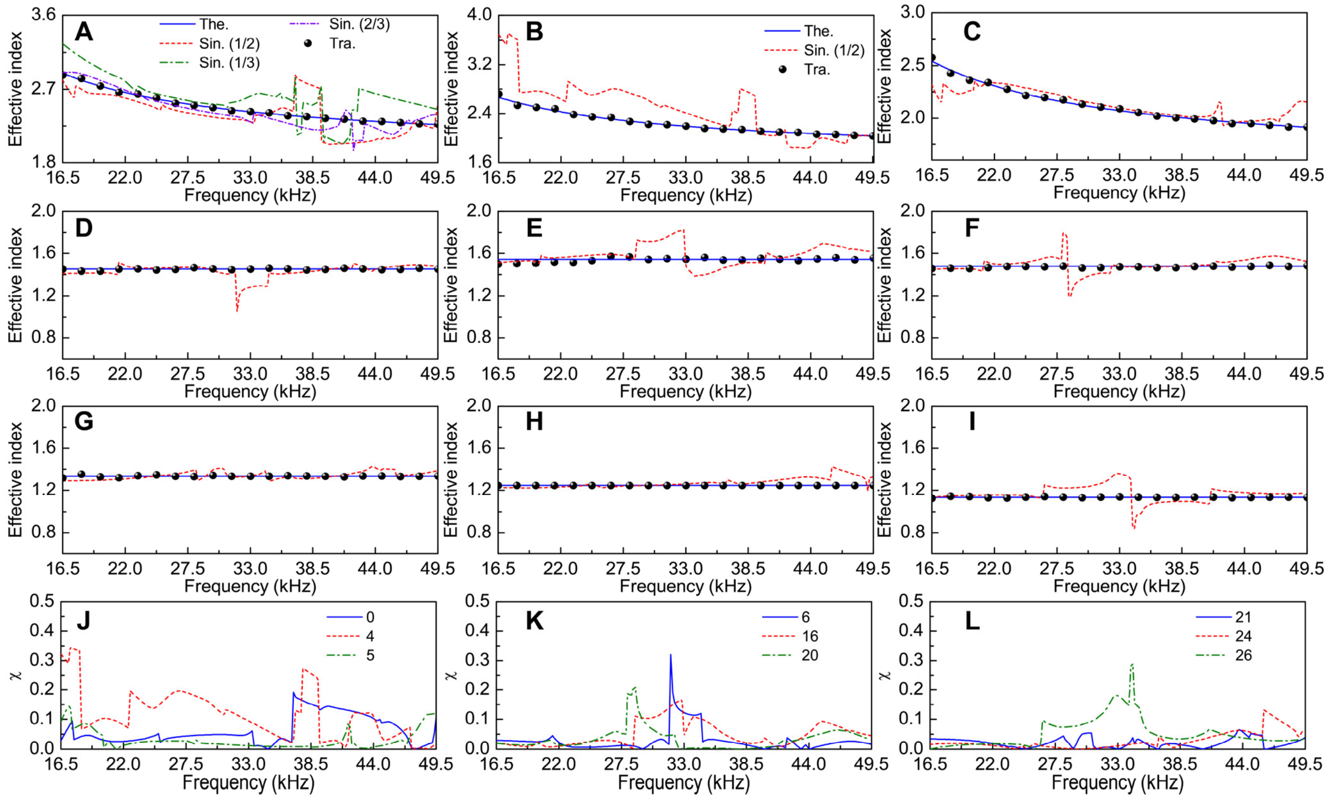

Fig. S26. Evidence of multiple scattering in the inversely designed metasurface for ultra-broadband levitation. a-i, Results show the comparison between the theoretical (The.), traditional retrieved (Tra.) and single-scattering (Sin.) induced effective indices of optimized Elements #3 (a), #4 (b), #5 (c), #6 (d), #16 (e), #20 (f), #21 (g), #24 (h) and #26 (i) Fig. S21, respectively. The index from the single-scattering approximation is calculated based on the division at location 1/2 shown in Fig. S4. **j-l**, Multiple-scattering factor of optimized Elements #3-#5 (j), #6, #16, #20 (k), #21, #24, #26 (l) in Fig. S21.

S14. Supplementary results for ultra-broadband levitation

To demonstrate the correctness of the optimized elements in Figs. S21 and S22, we construct a 3D inversely designed metasurface illustrated in Fig. 3a of the main text to systematically explore its wavefront manipulation within a broadband range. Compared with the theoretical ultrasonic levitation field in Fig. S27a, the 3D inversely designed metasurface can effectively induce the bottle beam to levitate an object within an ultra-broadband range. The only drawback is that the levitation position has a small deviation from the theoretical location. Nevertheless, the metasurface can lead to the ultra-broadband levitation near the same place with the exceeding 100% relative bandwidth, which is unprecedented and far better than the reported single-frequency or narrow-band ultrasonic levitation. Generally, the effect of the thermal-viscous loss [14] on the ultrasonic sound propagation in Fig. S27c is more remarkable than on the audible sound in Figs. S13 and S20. Meanwhile, the ultra-broadband levitation is not affected by the thermal-viscous loss. When considering the viscous loss of 0.01, the levitation fields in Fig. S27d can well match with those without loss in Fig. S27b. We would argue that, although losses in the metasurface are inevitable, thus leading to some adverse effects such as impaired transmission at some frequencies and rough boundaries, the targeted levitation can still be achieved. In ultrasonic experiments, the metasurface is directly placed on the ultrasonic transducers to guarantee sufficient transmitted acoustic energy. The space covered by the metasurface can show the effective required levitation fields, see Fig. S27e, demonstrating the robustness of the inversely designed metasurface.

If the thermal-viscous loss can be considered in topology optimization, it is expected to generate a better metasurface with ideal phase distribution and higher transmission. Of course, this would present greater challenges to the topology optimization.

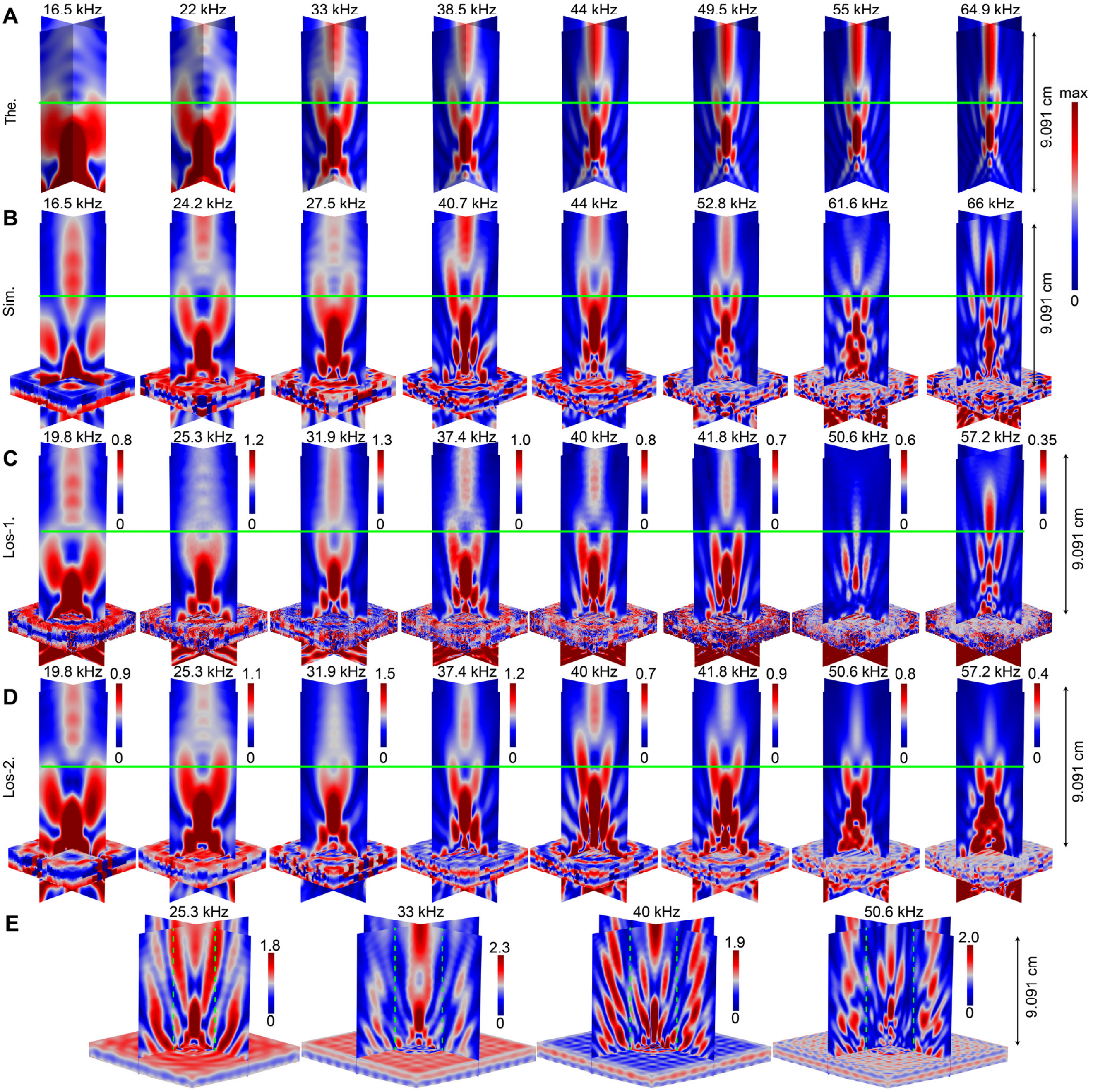

Fig. S27. Theoretical, simulated and measured levitation. a, Theoretical (The.) field considering the perfect phase distribution and the ideal total-transmission materials. **b**, Simulated (Sim.) acoustic amplitude fields without the loss within [16.5 kHz, 66 kHz] based on the inversely designed metasurface in Fig. 3. The green solid line indicates the desired levitation location (F_0=4.545 cm). **c**, Simulated acoustic amplitude fields with the thermal-viscous loss (Los-1.) or viscous loss of 0.01 (Los-2.). **d**, Acoustic amplitude profiles without the loss, with the thermal-viscous loss and with the viscous loss of 0.01 for the same horizontal line located at the theoretical focusing position F_0=4.545 cm. **e**, Simulated acoustic amplitude fields in which the inversely designed metasurface is directly placed on the ultrasonic source array with 12×12 ultrasonic transducer

with diameter of 10 mm. Every transducer acts a point source in simulation. The region inside the green dashed lines represents the area covered by the metasurface.

Two 324-element (18*18 elements) phased array transducers were assembled in this work, one is 32 kHz center frequency (T32-16, 16 mm diameter, Shanghai Nicera Sensor Co., Ltd, China) and the other is 40 kHz center frequency (T4010A1, 10 mm diameter, Nippon Ceramic Co., Ltd, Japan). The distance between the adjacent elements in 32 KHz array and 40 KHz array were 16.45 mm and 10.25 mm, respectively. An ultrasound system was fabricated to drive the array transducer, and the schematic of the system is shown in Fig. S28. The waveform in each element of the array can be controlled individually, and the minimal phase adjustment is 20 ns because the clock in FPGA is 50 MHz, which renders about 0.29-degree phase resolution for 40 kHz ultrasound, and about 0.23-degree phase resolution for 40 KHz ultrasound. The phase data were calculated on a computer first according to the trapping algorithms, and then the data were transmitted to a field-programmable gate array (FPGA) (Cyclone-V 5CGXFC7D7F31C8N, Altera Corporation, San Jose, CA) in driving board through an USB3.0 interface (CYUSB3014, Cypress, San Jose, CA). The FPGA processed the data and adjusted the phase of waveform in each channel accordingly. Finally, the waveforms were amplified by push-pull amplifiers, where the maximum amplitude of the waveforms can be as high as 28V, and the amplitude can be adjusted by controlling the power supply of the amplifiers.

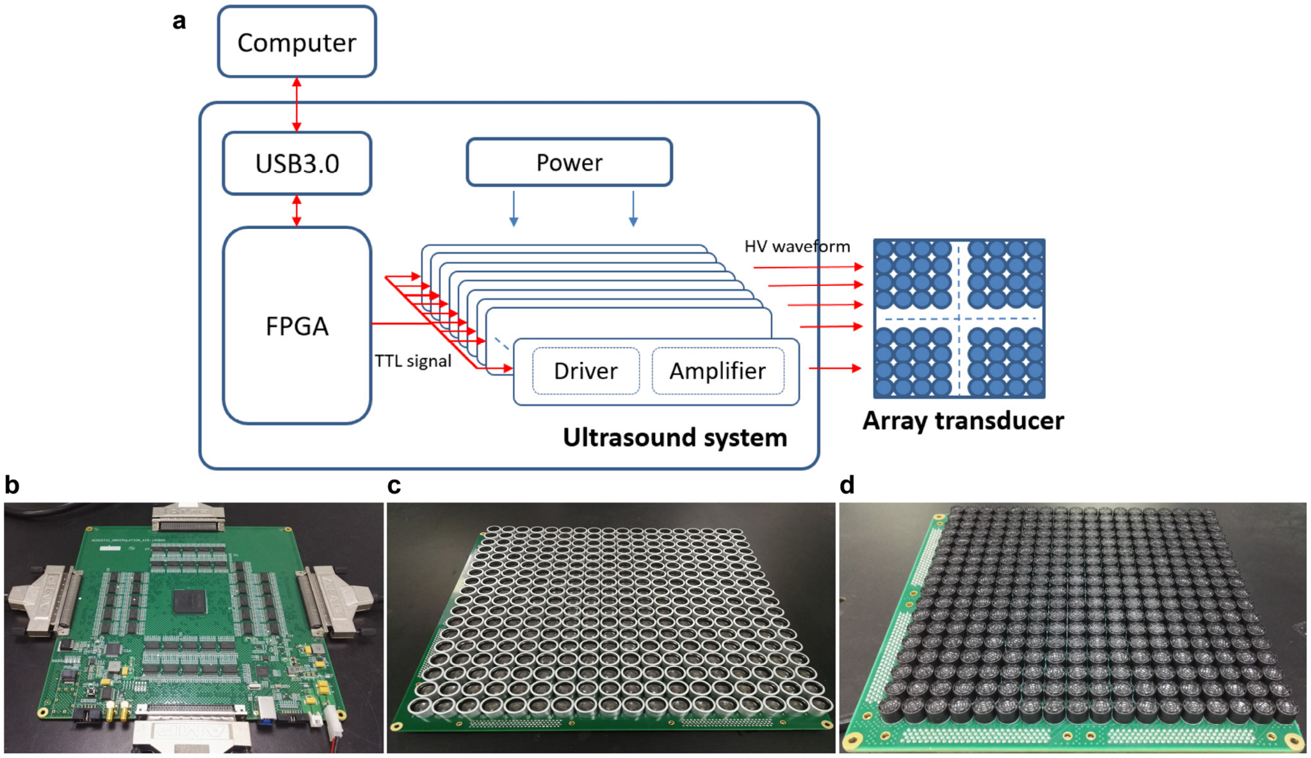

Fig. S28. Ultrasound array system and array transducers. a, The whole ultrasound system. **b**, Array system. **c**, Array transducer at 32 kHz. **d**, Array transducer at 40 kHz.

S15. Complex energy transport of representative optimized elements

To further clarify the underlying physical mechanism, we investigated the acoustic intensity distributions of the elements at some characteristic frequencies. As shown in Fig. 29(a) below, due to the superposition of multiple internal resonance modes, the acoustic intensity increases significantly in curved channels at different frequencies, which play a key role in the ultra-broadband phase compensation. Figure 29(b) shows that a

similar topology results in a similar feature in terms of energy flow as in Fig. 29(a). The difference is that the area with increased intensity gets larger. As shown in Fig. 29(c), the acoustic intensity not only increases in several local areas but also flows in the opposite direction. These features of energy flow differ from traditional space-coiling and Helmholtz-resonator structures.

Hence, it is the amplification and complex acoustic intensity flux path that creates ultra-broadband phase compensation and high transmittance.

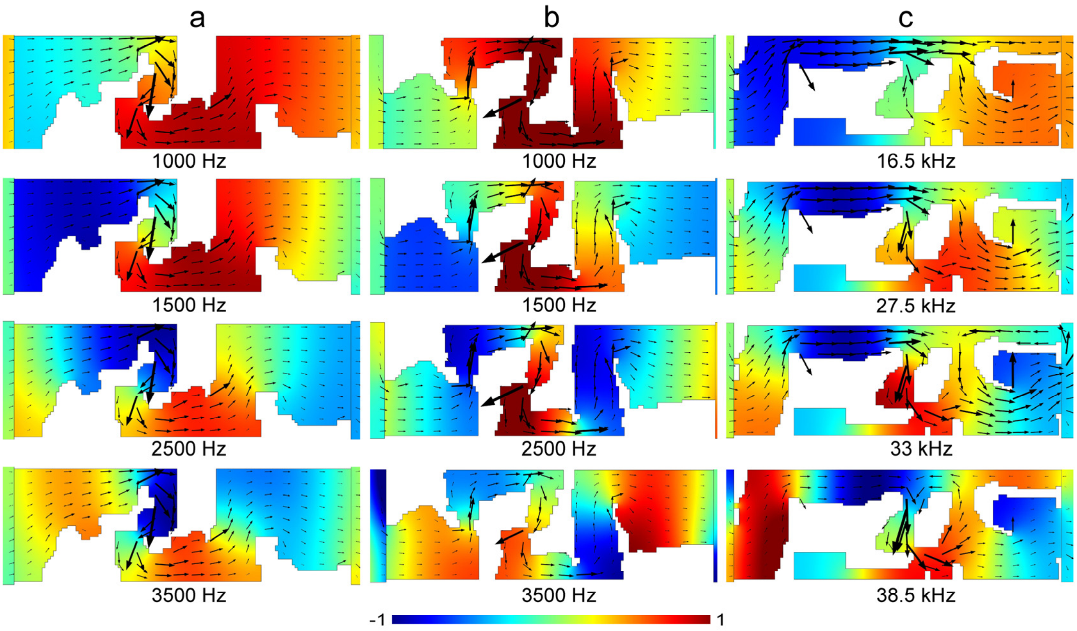

Fig. S29. Distribution of acoustic intensity of elements at characteristic frequencies. (a) Element #6 for beam deflection; (b) Element #7 for focusing; and (c) Element #0 for levitation. The arrow represents the flow of energy.

S16. Criterion of setting sampling frequencies for ultra-broadband metasurfaces

In principle, the way to select the discrete frequencies in designing the metasurfaces on demand is a crucial step, especially for broadband and even ultra-broadband metasurfaces. To elaborate this point, we take several optimization results of element #16 for levitation to determine the sampling frequencies within the target frequency range, see Fig. S30. Optimizations with 21, 11, 5 and 3 uniformly distributed sampling frequencies are performed to compare the resulting optimized topologies and their corresponding effective indices. Obviously, the case with 21 sampling frequencies is very close to the theoretical result within the target range. Meanwhile, the anastomotic effect of 21 sampling frequencies can be extended to the high-frequency range to achieve a wider bandwidth than the target one. However, the optimizations with 11, 5 and 3 sampling frequencies result in the different levels of deviation of effective indices. In addition, four optimizations give rise to the completely different topologies. This emphasizes the importance of the number of sampling frequencies on the topology optimization. Generally, less sampling frequencies will induce more local divergences. For the case with 11 frequencies, only the index around 33 kHz is different from the theoretical one. This phenomenon can usually be neglected in the design. In principle, more sampling frequencies can lead to more exact ultra-broadband property, whereas, alongside a higher computation cost.

Based on the abundant optimization studies, we propose a criterion for setting sampling frequencies for broadband metasurfaces, i.e, the minimal relative uniform frequency increment for a target frequency range should be 0.05. In view of the non-dispersive nature for ultra-broadband beam deflection and focusing, the

relative frequency increment can be relaxed to 0.1. This criterion can guide not only the inverse design but also the artificial and empirical design of metasurfaces.

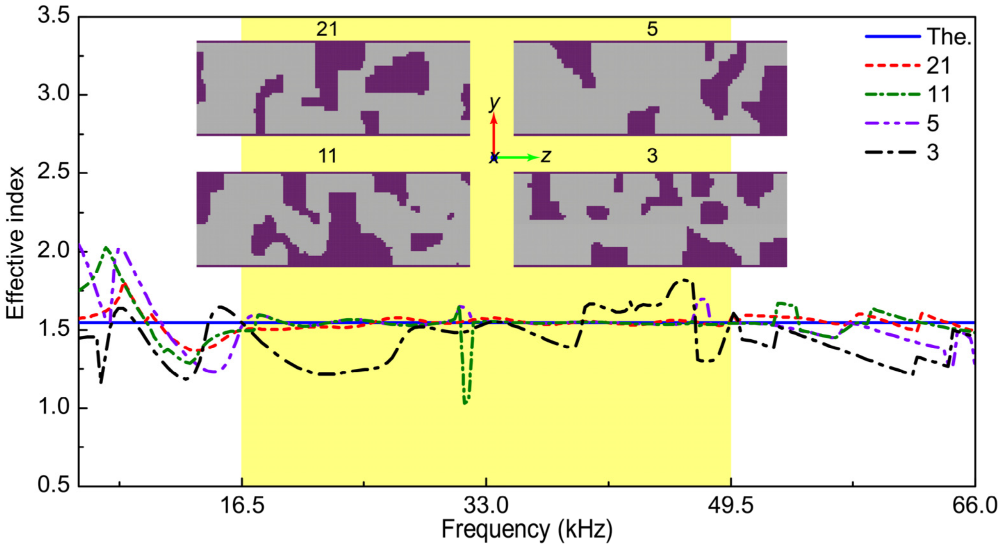

Fig. S30. Effective indices of the optimized elements under different numbers of the sampling frequencies for representative element #16 in Fig. S21. In topology optimization, 21, 11, 5 or 3 sampling frequencies are uniformly distributed within the target frequency range of [16.5 kHz, 49.5 kHz]. The required theoretical effective index (The.) is also presented. Subgraphs illustrate the topologies of the optimized elements under four different optimization cases. The yellow shadow area depicts the target frequency range in topology optimization. The purple and grey parts represent the solid and air elements, respectively.

S17. Analogical optical ultra-broadband metasurfaces

In view of the similarity between the electromagnetic and acoustic wave equations, the present inversely designed acoustic metasurfaces can be easily analogized to the optical metasurfaces by only selecting the suitable electromagnetic material parameters. As an example, we directly utilize the geometry of the inversely designed metasurface in Fig. 3 to design an optical metasurface, as displayed in Fig. S31. Indeed, the analogized optical metalens can also realize the ultra-broadband optical focusing with the relative bandwidth of about 120% within [0.874 GHz, 3.495 GHz], which is promising in the field of optical imaging [50]. Furthermore, this ultra-broadband achromatic feature demonstrates the robustness of the optimized metasurface topologies and revealed mechanisms once again.

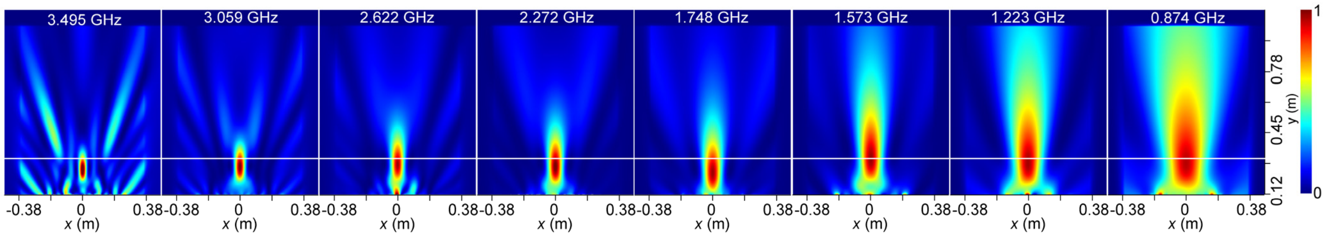

Fig. S31. Ultra-broadband achromatic optical metalens. Simulated magnetic amplitude fields at 8 representative frequencies within [0.874 GHz, 3.495 GHz]. The topology of the optical metasurface is directly copied from the inversely designed metasurface in Fig. 3. The outer region of the simulation model is set as the perfectly matched layer to avoid boundary reflection. Electromagnetic simulations consider the metasurface composed of metallic grating and air.

S18. Significance of broadband ultrasound levitation

The three representative applications explored in the paper require a broadband achromatic property to some extent. Broadband achromatic beam deflection can result in stable directional acoustic energy radiation and noise shielding. Broadband achromatic acoustic focusing enables high-definition medical ultrasound images and high-intensity energy capture. Broadband achromatic ultrasound levitation allows precise manipulation of particles with different weights and sizes. Without broadband feature, the metasurface-based technology is unlikely to meet the need for frequency-domain design. Moreover, the broadband passive structures we offer are noticeably superior to active tunable structures. For instance, our broadband metasurfaces do not require external field adjustment and are effective for signals with complex frequency content over a wide frequency range. In addition, the presented passive broadband achromatic metasurfaces can be suitable for manipulating wave packets, which is unlikely to be offered by active tunable ones.

For levitation of an object enclosed on a surface under the action of ultrasound, the strength of acoustic radiation in the second-order approximation can be determined by the equation

$$F = \frac{\omega}{2\pi}\int_S\int_0^{2\pi/\omega}\left[\left(\frac{\rho_0(\mathbf{v}\cdot\mathbf{v})}{2} - \frac{p^2}{2\rho_0 c_0^2}\right)\mathbf{n} - \rho_0\left(\mathbf{v}\cdot\mathbf{n}\right)\mathbf{v}\right]dtdS, \quad (23)$$

where ω is the operating frequency; S denotes the surface surrounding the object of levitation; $\mathbf{v}$ is the particle velocity; p represents the acoustic pressure; ρ_0 and c_0 are the density and sound velocity of the air, respectively; and $\mathbf{n}$ is the outward normal to the surface S. Obviously, the acoustic radiation force depends on the operating frequency. For the same enclosing surface, a higher frequency leads to a larger radiation force. Using the same force, a higher frequency requires a small enclosing object surface. This means that the broadband ultrasound levitation can handle particles of different weights and sizes. The corresponding schema is illustrated in Fig. S32.

Therefore, we believe broadband levitation using a passive metasurface has both scientific and practically implications. Moreover, our study offers the demonstration of ultra-broadband, stable and single-sided ultrasound levitation using metasurfaces.

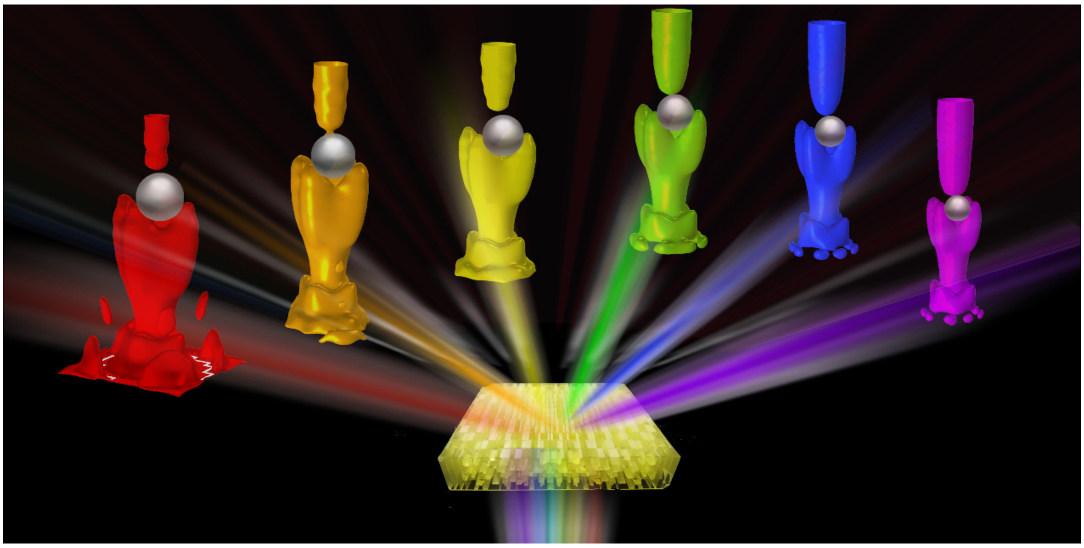

Fig. S32. Scheme of levitation of various particles by a broadband achromatic acoustic metasurface. The color changing from red to purple represents the increase of operating frequency.

S19. Supplementary Movies for ultra-broadband ultrasound levitation

To verify the achieved ultra-broadband bottle beam, the corresponding Supplementary Movie 1 is presented to vividly show the acoustic levitation at 40 kHz originating from the customized phase manipulation of the inversely designed 3D metasuraface in Fig. S21. Moreover, Supplementary Movie 2 can further demonstrate the very stable performance of the ultrasound levitation at 40 kHz. It should be emphasized here that the polystyrene bead can always suspend in the mid-air in the absence of external disturbance such as the air-flow and vibration. For comparison, Supplementary Movie 3 shows the response of the same polystyrene bread, put over the surface of the same ultrasonic source, when the metasuaface is absent. The polystyrene bead is directly washed away once the ultrasonic source is powered on. The same demonstration is provided in Supplementary Movies 4 and 5 to clearly show the very stable acoustic levitation at another operating frequency of 32 kHz.

S20. Supplementary Movies for evolutions of representative optimized elements

To illustrate the origin of the metasurface elements, we present some examples in Supplementary Movies 6–8 to show the evolution of three representative optimized elements for ultra-broadband beam deflection in Figs. S7a–c.

Movie S1. Ultrasonic levitation at 40 kHz originating from the customized phase manipulation of the inversely designed 3D ultra-broadband metasuraface.
Movie S2. Very stable ultrasonic levitation at 40 kHz using the inversely designed 3D ultra-broadband metasuraface.
Movie S3. Response of the same polystyrene bread putting over the surface of the same ultrasonic source, when the metasuaface is absent.
Movie S4. Oblique view of the very stable ultrasonic levitation at 32 kHz using the inversely designed 3D ultra-broadband metasuraface.
Movie S5. Front view of the very stable ultrasonic levitation at 32 kHz using the inversely designed 3D ultra-broadband metasuraface.
Movie S6. Evolution of optimized Element #2 for the ultra-broadband beam deflection from a random initial design.
Movie S7. Evolution of optimized Element #4 for the ultra-broadband beam deflection from an "Air" design.
Movie S8. Evolution of optimized Element #5 for the ultra-broadband beam deflection from an "Air" design.

S21. Simulations and experiments of ultra-broadband wave functionalities

To verify the desired ultra-broadband beam deflection, focusing and levitation, we investigate the full-wave propagation through assembling inversely designed metasufaces by using the acoustics module in the commercial software COMSOL Multiphysics 5.3. The background medium is air and the solid metasurfaces are treated as rigid material, so the acoustically-hard boundaries are used on the surface. Plane wave radiation boundary condition is adopted on the outer boundaries of the computational domain to eliminate the reflected waves, and the plane wave is excited on the incident port. To consider the thermal-viscous loss in the simulations, the thermoacoustics interface is used to compute the variations of the acoustic pressure, velocity, and temperature. The interface is required to accurately calculate the acoustic field in geometries of small dimensions, so the thermoacoustics domain is introduced in the region with element-cells containing complex acoustic channels. We then introduce the acoustic-thermoacoustic boundary nodes to couple the thermoacoustic domain to the acoustic domain. Viscosity and thermal conduction near the hard walls become important because they create a viscous and thermal boundary layer where losses are significant. Therefore,

the boundary layers are meshed with a dense element distribution in the normal direction along specific boundaries. The plane wave radiation boundary condition is also adopted on the outer boundaries of the simulation domains.

In the acoustic experiments on beam deflection and focusing, we used the same platform (Fig. S20e) to measure the transmitted acoustic fields. A loudspeaker array, located 0.15 m away from the input interface of the metasurface surrounded by sound-absorbing sponges, was used as the incident plane waves; while a mounted microphone was connected to the B&K device (Type 3160-A-042) to measure the acoustic pressure by moving in the 40 cm×50 cm scanning area. The measured signals at each position were averaged over four measurements to reduce the effect of noise. Using the Fourier transform, the whole acoustic filed was obtained after the scanning measurement.

In the ultrasonic experiments on levitation, we use a self-developed ultrasound array system (Fig. S28) to generate the ultrasonic plane waves, impinging on and propagating through the inversely designed metasurface. The amplitude and the phase of every ultrasonic transducer was set to be the same as before for plane wave generation. Since the metasurface can cover about 5×5 elements, only 12×12 or 10×10 ultrasonic transducers were open for the present ultrasonic experiments with the voltage of 20.3 V. Upon deploying the ultrasound array system, we put a polystyrene bead around the desired levitation position. As a result, the polystyrene bead was suspended in mid-air by the ultrasonic radial force. To characterize the ultrasonic field, the mounted microphone connected the B&K device (Type 3160-A-042) measured the acoustic pressure by moving within a 3D scanning area. The signals at each position were obtained out of four measurements. Using the Fourier transform, the whole acoustic filed was obtained after the scanning measurement.

References

1. Song BH and Bolton JS. A transfer-matrix approach for estimating the characteristic impedance and wave numbers of limp and rigid porous materials. *J Acoust Soc Am* 2000; **107**: 1131-1152.
2. Lucian Z, Popa BI and Starr AF *et al*. Design and measurements of a broadband two-dimensional acoustic metamaterial with anisotropic effective mass density. *J Appl Phys* 2011; **109**: 054906.
3. Khorasaninejad M, Chen WT and Devlin RC *et al*. Metalenses at visible wavelengths: Diffraction-limited focusing and subwavelength resolution imaging. *Science* 2016; **352**: 1190-1194.
4. Aieta F, Kats MA and Genevet P *et al*. Multiwavelength achromatic metasurfaces by dispersive phase compensation. *Science* 2015; **347**: 1342-1345.
5. Zhang P, Zhu TJ and Zhu X *et al*. Generation of acoustic self-bending and bottle beams by phase engineering. *Nat Commun* 2014; **5**: 4316.
6. Marzo A, Seah SA and Drinkwater BW *et al*. Holographic acoustic elements for manipulation of levitated objects. *Nat Commun* 2015; **6**: 8661.
7. Baresch D, Thomas JL and Marchiano R *et al*. Observation of a single-beam gradient force acoustical trap for elastic particles: acoustical tweezers. *Phys Rev Lett* 2016; **116**: 024301.
8. Sieck CF, Alù A and Haberman MR *et al*. Origins of Willis coupling and acoustic bianisotropy in acoustic metamaterials through source-driven homogenization. *Phys Rev B* 2017; **96**: 104303.
9. Koo S, Cho C and Jeong JH *et al*. Acoustic omni meta-atom for decoupled access to all octants of a wave parameter space. *Nat Commun* 2016; **7**: 13012.
10. Muhlestein MB, Sieck CF and Wilson PS *et al*. Experimental evidence of Willis coupling in a one-dimensional effective material element. *Nat Commun* 2017; **8**: 15625.
11. Fokin V, Ambati M and Sun C *et al*. Method for retrieving effective properties of locally resonant acoustic metamaterials. *Phys Rev B* 2007; **76**: 144302.
12. Cummer SA, Christensen J and Alù A. Controlling sound with acoustic metamaterials. *Nat Rev Mater* 2016; **1**: 166001.
13. Sieck CF, Alù A and Haberman MR, Origins of Willis coupling and acoustic bianisotropy in acoustic metamaterials through source-driven homogenization. *Phys Rev B* 2017; **96**: 104303.

14. N. J. Gerard, Y. Li and Y. Jing. Investigation of acoustic metasurfaces with constituent material properties considered. *J Appl Phys* **123**, 124905 (2018).
